# Bioresorbable, wireless, and battery-free system for electrotherapy and impedance sensing at wound sites

**DOI:** 10.1126/sciadv.ade4687

**Published:** 2023-02-22

**Authors:** Joseph W. Song, Hanjun Ryu, Wubin Bai, Zhaoqian Xie, Abraham Vázquez-Guardado, Khizar Nandoliya, Raudel Avila, Geumbee Lee, Zhen Song, Jihye Kim, Min-Kyu Lee, Yugang Liu, Mirae Kim, Huifeng Wang, Yixin Wu, Hong-Joon Yoon, Sung Soo Kwak, Jaeho Shin, Kyeongha Kwon, Wei Lu, Xuexian Chen, Yonggang Huang, Guillermo A. Ameer, John A. Rogers

**Affiliations:** ^1^ Department of Biomedical Engineering, Northwestern University, Evanston, IL, USA.; ^2^ Querrey Simpson Institute for Bioelectronics, Northwestern University, Evanston, IL, USA.; ^3^ Center for Advanced Regenerative Engineering, Northwestern University, Evanston, IL, USA.; ^4^ Department of Advanced Materials Engineering, Chung-Ang University, Anseong, Korea.; ^5^ Department of Applied Physical Sciences, The University of North Carolina at Chapel Hill, Chapel Hill, NC, USA.; ^6^ State Key Laboratory of Structural Analysis for Industrial Equipment, Department of Engineering Mechanics, Dalian University of Technology, Dalian 116023, P. R. China.; ^7^ DUT-BSU Joint Institute, Dalian University of Technology, Dalian 116024, China.; ^8^ Department of Mechanical Engineering, Northwestern University, Evanston, IL, USA.; ^9^ Department of Electronic Engineering, Gachon University, Seongnam 13120, Korea.; ^10^ Center for Bionics of Biomedical Research Institute, Korea Institute of Science and Technology, Seoul, Korea.; ^11^ Department of Electrical Engineering, Korea Advanced Institute of Science and Technology, Daejeon, Korea.; ^12^ Academy for Advanced Interdisciplinary Studies, Peking University, Beijing, 100871 China.; ^13^ Departments of Civil and Environmental Engineering, Northwestern University, Evanston, IL, USA.; ^14^ Department of Materials Science and Engineering, Northwestern University, Evanston, IL, USA.; ^15^ Simpson Querrey Institute for Bionanotechnology, Evanston, IL, USA.; ^16^ Chemistry of Life Processes Institute, Northwestern University, Evanston, IL, USA.; ^17^ Department of Surgery, Feinberg School of Medicine, Northwestern University, Chicago, IL, USA.; ^18^ International Institute for Nanotechnology, Northwestern University, Evanston, IL, USA.; ^19^ Department of Neurological Surgery, Feinberg School of Medicine, Northwestern University, Evanston, IL, USA.

## Abstract

Chronic wounds, particularly those associated with diabetes mellitus, represent a growing threat to public health, with additional notable economic impacts. Inflammation associated with these wounds leads to abnormalities in endogenous electrical signals that impede the migration of keratinocytes needed to support the healing process. This observation motivates the treatment of chronic wounds with electrical stimulation therapy, but practical engineering challenges, difficulties in removing stimulation hardware from the wound site, and absence of means to monitor the healing process create barriers to widespread clinical use. Here, we demonstrate a miniaturized wireless, battery-free bioresorbable electrotherapy system that overcomes these challenges. Studies based on a splinted diabetic mouse wound model confirm the efficacy for accelerated wound closure by guiding epithelial migration, modulating inflammation, and promoting vasculogenesis. Changes in the impedance provide means for tracking the healing process. The results demonstrate a simple and effective platform for wound site electrotherapy.

## Teaser

Wireless, biodegradable and battery-free electrotherapy system enables remote accelerating and monitoring wound healing process.

## INTRODUCTION

Diabetes mellitus is a major public health problem that imposes substantial productivity and financial burdens on society, with health care costs in the United States exceeding $327 billion annually and projected to increase at a rate of 1 billion per year and contributing to the increase in years lived with disability (*1*). One of the severe complications of the approximately 30 million people living with diabetes in the United States is diabetic foot ulcers (DFUs), which occur in 15 to 25% of patients with diabetes (*2*). If not appropriately treated, then these and other types of chronic wounds may lead to amputations. Diabetic-related complications with chronic wounds such as DFUs are the number one cause of nontraumatic lower limb amputations worldwide (*3*). Although wound care management is well established, the multifactorial etiology, patient-specific circumstances, high regulatory and market barriers to entry, adoption for new biologics-based therapies, and adequate access to care remain challenges to effective treatment (*4*). Therefore, research into new strategies and associated technologies that prevent or improve the outcome of chronic DFUs must be developed.

A variety of strategies have been investigated to address the problems that contribute to chronic DFUs, such as impaired angiogenesis, reduced dermal cell migration and proliferation, excessive oxidative stress, prolonged inflammation, and bacterial infection (*5*). Methods include the release of drugs and biologics at the wound (*6*), the use of bioactive materials as dressings (*7*), cell transplantation (*8*), tissue-engineered or skin equivalent products (*9*), the use of vacuum (*10*), and electrotherapy (*11*–*13*). Many of the experimental approaches show promise in preclinical models and some clinical trials; nevertheless, they face notable regulatory, manufacturing, user adoption hurdles, and high development costs. Products that are in clinical use can be too expensive for widespread application, as in the case of biologics, and/or they do not fully address the underlying problems that contribute to chronic wounds. An additional unmet need is in capabilities to monitor the status of the wound to better inform clinical decisions and improve the effectiveness of therapies.

Electrotherapy has been used and investigated as a method to accelerate the closure of skin wounds (*11*, *12*). Related electrical approaches may also enable simultaneous monitoring of wound status (*13*). The hypothesis is that applied electric fields restore endogenous wound currents to recapitulate the natural healing mechanism. Although case studies suggest that electrostimulation is effective in wound closure, its use is not widespread in clinical practice. Reasons for this limited adoption include lack of understanding of the optimal settings (e.g., for dosing, timing, and type of electrical stimulation), inadequate form factors in the hardware (e.g., use of bulky equipment that requires inpatient care and leads to decreased patient compliance), and poor control interfaces with cumbersome modes of use (e.g., the treatment often must be applied daily). A dominating additional concern for any type of therapeutic or diagnostic device that requires direct physical interfaces with the wound site is in the potential for damage to fragile soft tissues during removal after a period of use (*14*, *15*).

Here, we introduce a bioresorbable, wireless, and battery-free electrotherapy system (BES) that provides electrostimulation and impedance measurements across a wound in a manner that avoids the aforementioned disadvantages. The stimulation process mimics naturally occurring endogenous electric fields to promote healing. The impedance data provide the basis for real-time monitoring of wound closure. The BES includes a pair of compliant electrodes that supports stable operation over several weeks and then slowly bioresorbs via hydrolysis to eliminate the need for retrieval. A miniaturized wireless, battery-free electronics module interfaces to these electrodes, with a graphical user interface that runs on a smartphone. This technology, which can be easily used in both the hospital and home settings, has the potential to improve the care of patients with chronic DFUs and with further study may provide new options to treat other skin wounds.

## RESULTS

### Bioresorbable, wireless, and battery-free electrotherapy system

**Fig. 1.**
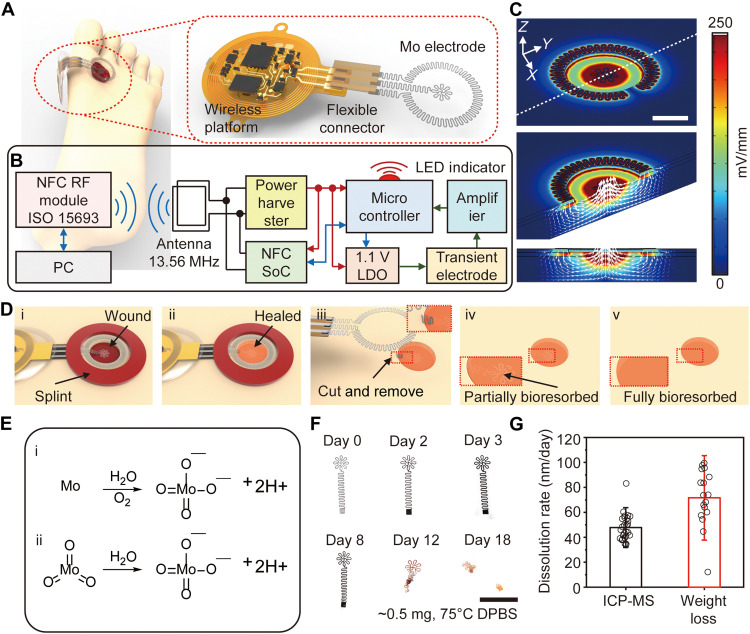
Materials and designs of a bioresorbable, wireless, and battery-free electrotherapy system. (**A**) Schematic illustrations of a transient, wireless, battery-free system for electrotherapy mounted on a wound on the foot (left) and in an enlarged view (right) that highlights the different components. (**B**) Operational diagram of the entire system. RF, radio frequency; ISO, interconnection system operation; LDO, low-dropout regulator. (**C**) FEA results of the electric field between the positive (+) and negative (−) electrodes. Scale bar, 3 mm. (**D**) Schematic illustrations of the mode of use, device on a wound (i) before and (ii) after healing, (iii) removed by cutting the traces to the anode, (iv) partially bioresorbed after a period of therapy, and (v) fully bioresorbed; the semitransparent orange color represents the healed skin. (**E**) Chemical reactions that govern the reaction of Mo and MoO_3_ with water. (**F**) Photographs of dissolution of an inner electrode structure with interconnects, captured at different times of immersion in Dulbecco’s phosphate-buffered saline (DPBS; pH 7.4) at 75°C. Scale bar, 5 mm. (**G**) Dissolution rates for a Mo electrode calculated by inductive coupled plasma mass spectrometry (ICP-MS) analysis of dissolved Mo ions (*n* = 9, individual samples) and for a Mo foils determined by weight loss (*n* = 4, individual samples).

Figure 1A shows a schematic illustration of a BES on the surface of a wound on the foot. The right side of this frame highlights the various components, including a wireless platform, a releasable flexible connector, and a stimulator that consist of a concentric pair of Mo electrodes with filamentary serpentine layouts for stretchability and without an encapsulation layer. The wireless platform consists of (i) a power harvesting coil that operates by magnetic inductive coupling at a resonance frequency of 13.56 MHz, to power the system, (ii) a near-field communication (NFC) system on chip that supports wireless communication, (iii) a red light-emitting diode (LED) that serves as a visual indicator of system operation, and (iv) a microcontroller unit that supplies controlled voltages for stimulation and measures the applied current (Fig. 1B and figs. S1 and S2). The electric current is measured with a shunt resistor, connected in series with the electrode. The voltage in the shunt resistor is amplified with an instrumentation amplifier and then digitalized with the microcontroller’s analog-to-digital converter (ADC). The current is then calculated considering the shunt resistor value, the ADC value, and the amplification gain. The wireless device uses a customized routine to perform sampling averaging (*n* = 10 to 20) on a chip. With this device option and the moderate low amplification gain set in the analog front-end module, the noise due to other factors becomes negligible. The inner and outer Mo electrodes reside at the center of the wound and slightly outside the wound around its perimeter, respectively. The thin geometries of these electrodes allow for mechanical flexibility; the serpentine layouts afford some level of stretchability. Specifically, the inner electrode consists of a serpentine trace with a thickness of 15 μm and a width of 120 μm in a flower-like design (*d*_in_; diameter of 2 mm); the outer electrode adopts a similar serpentine shape with similar thickness and width (*d*_out1_; inner diameter of 8 mm; *d*_out2_; outer diameter of 10.5 mm) (fig. S3). The flexible connector allows the wireless platform to be positioned onto healthy skin nearby the wound site.

Three-dimensional (3D) finite element analysis (FEA) captures the spatial distribution of the electric field, voltage, current density, and temperature during DC 1.1-V stimulation with the inner electrode on adipose tissue and the outer electrodes on the epidermis to mimic or reproduce in vivo conditions (Fig. 1C and figs. S4 and S5). The inward DC from the healthy site to the wounded area mimics naturally driven endogenous wound currents (*16*). The electric field strength approaches ~250 mV/mm near the inner electrode. Other regions of adipose tissue between the outer and inner electrodes experience field strengths of ~100 mV/mm, known to be sufficient to cause migration of human keratinocyte cells to accelerate wound healing processes (*17*, *18*).

Figure 1D illustrates the scheme for using this system in electrotherapy. A sutured splint ring structure fixes the Mo electrodes at the site of a wound, positioned as described above (Fig. 1D-i). As the wound heals, the inner Mo electrode becomes embedded in the regenerated skin (Fig. 1D-ii and fig. S6]. After completing the therapy, cutting the trace that leads to this electrode allows removal of the wireless platform and the outer Mo electrode (Fig. 1D-iii). The inner electrode (mass < 500 μg) gradually bioresorbs into nontoxic products (Mo + 2H_2_O + O_2_ → MoO_4_^2−^ + 4H^+^ and MoO_3_ + H_2_O → MoO_4_^2−^ + 2H^+^) to lastly disappear without a trace over a time scale of months [Fig. 1, D (iv and v) and E]. In vitro tests of the Mo electrodes indicate good biocompatibility (fig. S7). Figure 1F presents a sequence of images of the inner electrode structure at various points of dissolution in Dulbecco’s phosphate-buffered saline (DPBS; pH 7.4) at 75°C, corresponding to a roughly 16 times acceleration relative to body temperature. The electrode begins to lose its shape after day 12 and almost disappears after day 18. Inductive coupled plasma mass spectrometry (ICP-MS) analysis and weight loss measurements associated with dissolution of a foil of Mo (~1 cm^2^) indicate a dissolution rate of 50 to 70 nm/day at 37°C with various time points, which is consistent with previous reports and with the results of accelerated testing (Fig. 1G and fig. S8) (*19*, *20*).

### Dissolution of the thin, serpentine Mo electrodes

**Fig. 2.**
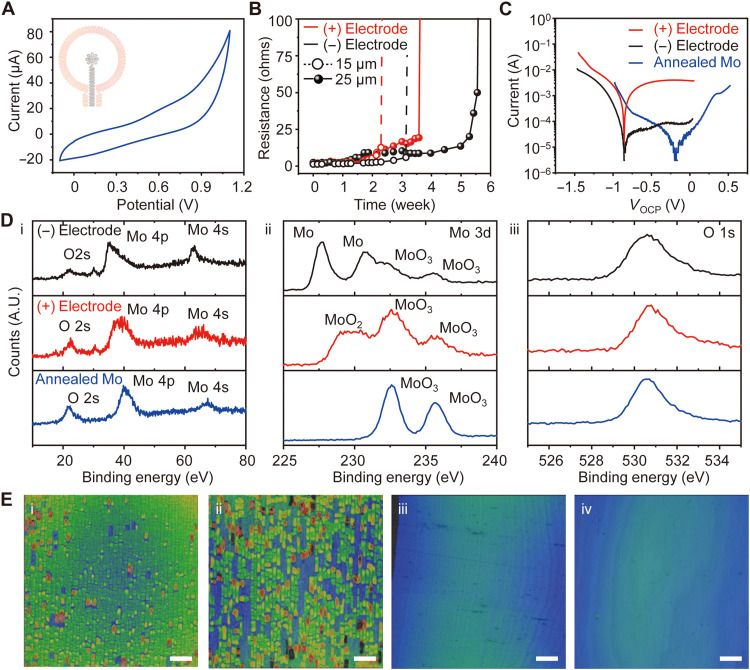
Electrical and physical properties of bioresorbable electrodes at physiological conditions. (**A**) Cyclic voltammogram of a pair of Mo electrodes as an electrical stimulator during immersion in DPBS (pH 7.4) at room temperature. The inset image represents the working electrode (red) and counter electrode (black). (**B**) Changes in resistances of Mo electrodes (positive and negative) with an applied voltage of 1.1 V for 30 min/day during immersion in DPBS (pH 7.4) at 37°C. (**C**) Potentiodynamic polarization curves for positive and negative Mo electrodes after 20 min of open circuit potential (OCP) measurement during immersion in DPBS at pH 7.4. (**D**) XPS data from the surfaces of the electrodes. (**E**) Optical surface profiles of different positions on a positive Mo electrode showing (i) dense Mo oxide flakes and (ii) partially exfoliated flakes. Blue indicates Mo; green and red indicate Mo oxide. Similar profiles for the (iii) negative electrode and (iv) pristine Mo. Scale bars, 100 μm. A.U., arbitrary units.

The electrical properties of the electrodes without an encapsulation layer and their stability of operation over a time frame set by the healing process have not been previously investigated and are important to understand for this potential medical application (*21*, *22*). Cyclic voltammetry (CV) analysis of a Mo electrode immersed in DPBS (pH 7.4) at room temperature indicates no additional redox response except normal oxidation and reduction of Mo across voltages from 0 to 1.1 V (Fig. 2A). CV analysis in an alkaline environment was similar to a pH of 7.4 (fig. S9). Tests of electrical degradation involve immersion in DPBS (pH 7.4) at 37°C housed in a container formed in poly(dimethylsiloxane) (PDMS; volume of 5 ml) with a polyimide top cover film to prevent water evaporation (fig. S10). DC stimulation causes electrolytic corrosion on the anode (+), thereby accelerating the rate of corrosion to values ~30% above those of the cathode (−), for the case of an applied DC voltage of 1.1 V for 30 min/day (Fig. 2B). Studies of Mo electrodes with thicknesses of 15 and 25 μm confirm that functional lifetime has a linear relationship with thickness. The rate of corrosion of the cathode in this case is similar to that of an electrode without applied voltage (fig. S11). Because of accelerated corrosion at the interface between the PDMS container and the Mo electrodes (fig. S12) (*23*), the results presented here overestimate the intrinsic rates of corrosion. The annealing process forms a thin and uniform layer of molybdenum trioxide (MoO_3_) on the Mo surface to suppress the initial dissolution rate due to relatively higher corrosion potential (−0.16 V_corr_) than that of Mo (Fig. 2, C and D). After this MoO_3_ passivation layer dissolves from the surface at an applied DC voltage of 1.1 V over 24 hours in DPBS at room temperature, the corrosion potentials of both anode and cathode decrease to −0.85 V_corr_. X-ray photoelectron spectroscopy (XPS) analysis indicates the formation of electrochemically grown molybdenum dioxide (MoO_2_) at the anode and reduction of Mo at the cathode (Fig. 2D). Optical profiler images of different anode surfaces show flakes of MoO_2_ and MoO_3_ that exfoliate from the Mo surface due to the high Pilling-Bedworth ratio [Fig. 2E (i and ii) and fig. S13] (*24*). This process exposes fresh Mo at the anode, thereby sustaining the original surface impedance over time (fig. S14). The cathode retains a clean surface.

### Wireless in vivo operation of the BES

**Fig. 3.**
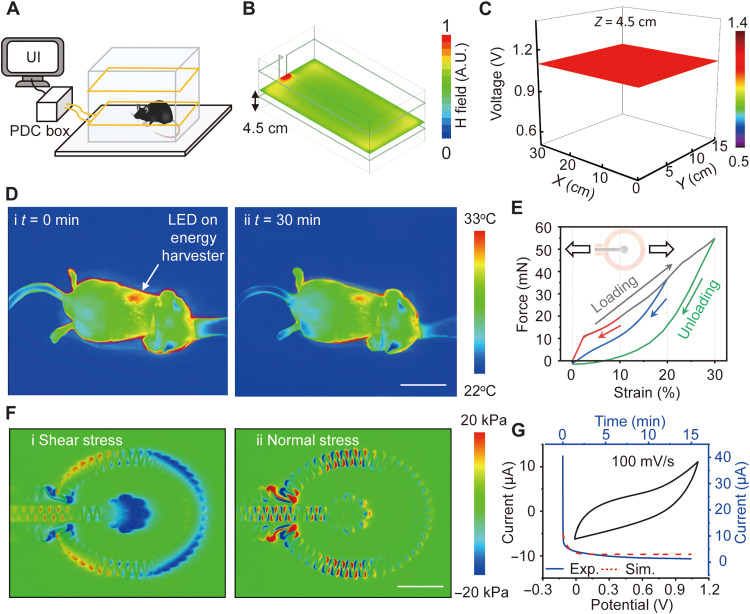
Characterization of thermal, mechanical, and electrochemical properties at in vivo conditions. (**A**) Schematic illustration of a wireless electrotherapy system for in vivo experiments. (**B**) Simulated electromagnetic field distribution at the central plane of a cage [dimensions, 30 cm (length) by 15 cm (width) by 15 cm (height)] surrounded by a double-loop antenna at heights of 3 and 9 cm. (**C**) Position-dependent stimulation voltage across the cage at a height of 4.5 cm. (**D**) Infrared images of temperature changes after operating the device on a mouse for (i) 0 min and (ii) 30 min. Scale bar, 10 mm. (**E**) Simulated strain-force curves associated with loading and unloading Mo electrodes over different ranges of strain, from 10, 20, and 30%. (**F**) Computed (i) shear and (ii) normal stress distributions on the skin. The color indicates stresses on the skin induced by stretching the electrodes by ~9%. Scale bar, 3 mm. (**G**) Cyclic voltammogram (100 mV/s, scan rate) and chronoamperometry curves of a Mo device for 15 min on the wound.

In vivo animal model studies use a cage with an inductive power transmitter coil wrapped around the structure to allow for wireless, battery-free operation without constraint on animal behaviors and movements. A graphical user interface operating on a separate computer supports real-time control over the stimulation parameters and serves as an interface to record the associated currents that pass through the anode and cathode. Simulations indicate that the transmitter coil establishes uniform electromagnetic fields at all regions except for the boundaries of the cage (Fig. 3B and fig. S15). When the height of the BES device is 4.5 cm, comparable to the position of the back of a mouse, the output voltage is 1.1 V, as actively controlled by linear and low-dropout regulators (Fig. 3C). Infrared imaging indicates no crucial heating associated with electrodes during stimulation (Fig. 3D and figs. S16 and S17). The surface of the wireless platform can reach temperatures of 33°C while on the animal (body temperature of 27°C). FEA simulations for the outer Mo electrode under uniaxial stretching show elastic behavior up to 9% stretching, corresponding to stretching of the skin in an isotropic manner to ~15% (Fig. 3E and fig. S18) (*25*). FEA simulations indicate that the shear and normal interfacial stresses in most areas remain below the threshold for sensation (~20 kPa) (*26*) for deformations of the skin to tensile strains of the Mo electrode up to ~9% (Fig. 3F). Stretching to 20 and 30% leads to plastic deformation of the Mo by strains of 1 and 7%, respectively. The strain in the electrode structures is ~0.2% at a bending radius of 4 mm or a torsion angle of 60° (fig. S18). In clinical cases, patients wear a total contact cast to eliminate pressures on the foot ulcers. Experimental testing shows that the Mo electrode was also stable under continuous frictional forces and pressures associated with walking, without the cast (fig. S19). The impedances between the electrodes when placed on the epidermis (case 1) and when the outer electrode is on the epidermis and the inner electrode is on the adipose tissue (case 2) are ~570 and ~200 kilohms at a frequency of 1 kHz, respectively. The impedance with both electrodes on adipose tissue (case 3; fig. S20) is ~100 times smaller than that of case 2. In vivo CV measurements with the Mo electrodes on the wound (case 2) across a voltage range from 0 to 1.1 V shows that Mo electrodes do not undergo additional redox reactions except normal oxidation and reduction of Mo when exposed to the epidermis, adipose tissue, or biofluids (Fig. 3G). Experimental results and simulations for the applied current between the inner and outer electrodes under a constant voltage of 1.1 V are similar with a capacitive response at the beginning, as expected on the basis of ionic transport, and then show a saturated DC current due to a normal redox reaction.

### Acceleration of wound closure rate and granulation tissue formation in diabetic mice

**Fig. 4.**
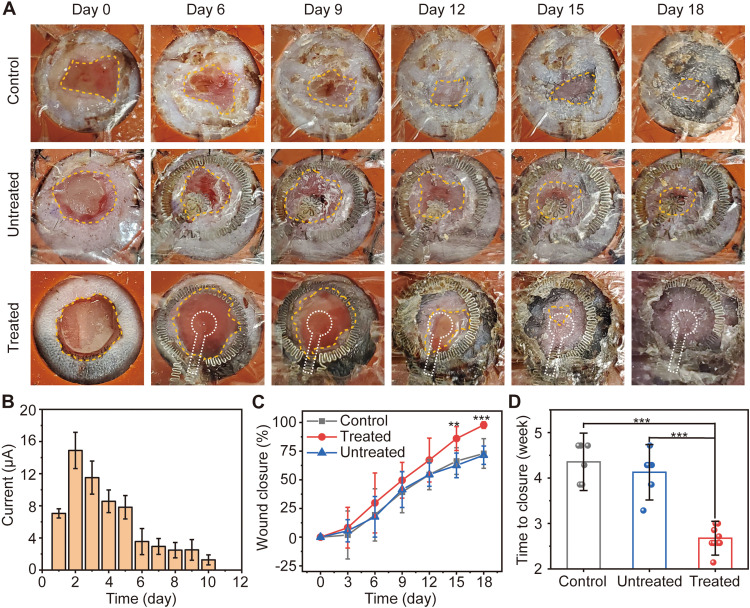
Electrostimulation accelerates wound closure in diabetic animals. (**A**) Digital images of wound closure. The yellow dotted line highlights the perimeter edges of the wound in each image. The inner negative electrode is embedded in the wound during the healing process and is overlaid by a white dot line. The inner diameter of splint is 10 mm. (**B**) Average detected current measurements correlate with wound closure in diabetic animals. (**C**) Quantification of wound closure for day 18 after wounding (9 ≤ *n* ≤ 11; **P < 0.01 and ***P < 0.001). (**D**) Summary of the complete wound closure times (5 ≤ *n* ≤ 11; ***P < 0.001).

We hypothesized that accelerated in vitro keratinocyte migration toward the anode (fig. S21) will translate to an enhanced wound healing response in vivo by reintroducing an endogenous electric field toward the center of the wound (fig. S6). Evaluations of wound area changes and wound closures serve as the basis for comparing the healing progress between control and electrostimulated groups (*27*). The splinted, full thickness excisional dermal wound model in diabetic mice minimizes wound healing due to skin contraction and enables a healing process that includes granulation and re-epithelialization, similar to the healing processes for wounds in the human skin (*28*). Figure 4A represents the healing progression of three different diabetic mouse groups, where the orange circle highlights the wound area (control, untreated, and treated mouse). The treated group involves Mo electrodes with continuous DC electrostimulation for 30 min every day until full wound closure; the untreated group involves Mo electrodes without electrostimulation; the control groups had no Mo electrode and no treatment other than a protective dressing. The electrodes must still be covered with a traditional dressing such as Tegaderm or similar to protect the wound site. The electrostimulation for 30 min/day until full wound closure was selected on the basis of previous clinical studies (*29*–*31*). The studies focus on two circular excisional wounds (6 mm in diameter) on the left and right sides of the back for the treated/untreated groups and the control group, respectively. Digital pictures of the wound collected every 3 days reveal the extent of wound closure at these time points.

Wound exudate formed as part of the inflammatory response (*32*) contains proteins, essential nutrients, etc., as a moist and electrically conductive environment on the wound. The amount of exudate decreases after the inflammatory and proliferation stages, and then the wound gradually dries. Reflecting these processes, after the full integration of the device into the surrounding, the current between the outside and inside Mo electrodes increases up to ~20 μA at the beginning of the inflammatory stage and gradually decreases to 0 when the wound is fully dry (Fig. 4B). The system can detect up to the point when the wound gets fully dry. As dryness is a key factor in wound healing (*33*), our system can accurately monitor critical early-stage healing. Benchtop tests with a hydrogel confirm that drying of the wound affects the sensed current (fig. S22). This drying causes the ion conductivity to decrease, leading to a reduction of the current to 0 μA. In this way, the current provides an estimate of the healing progress, as signature of which is drying of the wound. Experiments show that the current measured from normal mice decays faster (fig. S22) than that of diabetic mice, consistent with the expected relative rates of healing (*34*).

Electrostimulation using the devices and parameters reported here reduces the times for closure of excisional splinted wounds by ~30% compared to those of control and untreated groups. No significant weight changes occur during electrostimulation (fig. S23). In addition, the device itself does not change the wound healing rate, as determined by experiments performed without the electrostimulation treatment (9 ≤ *n* ≤ 11 per group; **P < 0.01 and ***P < 0.001; Fig. 4C and fig. S24). Wounds treated with electrostimulation heal at a significantly accelerated rate compared to other groups, such that 86.0 ± 10% closure occurs on day 15 compared to 62.6 ± 11% for the untreated and 66.4 ± 12% for the control groups. Most mice in the control (*n* = 11) and untreated groups (*n* = 5) require more than 4 weeks to complete wound closure. In comparison, closure occurs in the treated group (*n* = 11) in less than 3 weeks (***P < 0.001; Fig. 4D). Additional experiments use a simplified BES with only the electrostimulation function (fig. S25).

### Promotion of re-epithelialization and angiogenesis in wounds of diabetic animals

**Fig. 5.**
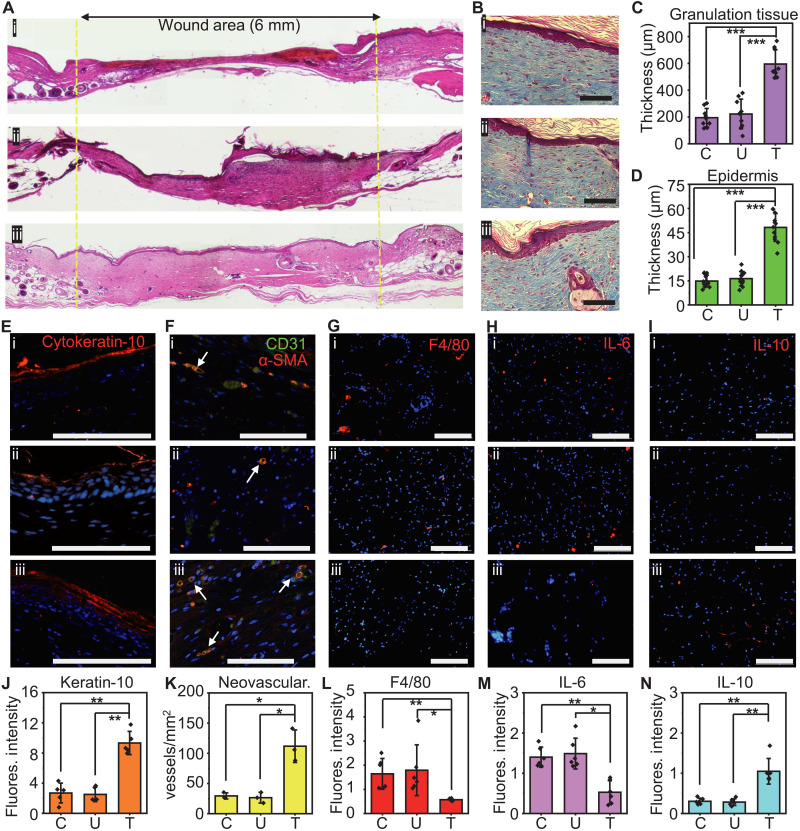
Electrostimulation facilitates proregenerative processes in the wound. (**A**) Digital images of H&E-stained tissue at day 18 after wounding. The yellow dashed line highlights the perimeter of the wound. (**B**) Masson’s trichrome–stained tissue at day 30 after wounding. Quantification of (**C**) the granulation tissue thickness at day 18 after wounding (**D**) the epidermis layer at day 30 after wounding. (**E**) Epithelial differentiation visualized by cytokeratin-10 immunofluorescence staining. (**F**) Angiogenesis visualized by CD31 and α-SMA immunofluorescence staining. Arrows point to the lumen of the newly formed vessels. (**G**) Macrophage marker visualized by F4/80 immunofluorescence staining. (**H**) A proinflammatory marker visualized by interleukin-6 (IL-6) immunofluorescence staining. (**I**) An anti-inflammatory marker visualized by IL-10 immunofluorescence staining. Quantification of (**J**) the keratin-10, (**K**) the neovascularization, (**L**) the F4/80, (**M**) the IL-6, and (**N**) the IL-10. (A, B, and E to I) (i), (ii), and (iii) indicate control, untreated, and treated groups, respectively. (J to N) C, U, and T represent control, untreated, treated groups, respectively. All data are represented as means ± SD. *P < 0.05, **P < 0.01, and ***P < 0.001. Scale bars, 100 μm (B and E to I).

Histological studies quantify the granulation tissue thickness, epithelial thickness, and keratin-10 signal. The results of hematoxylin and eosin (H&E) staining of the wounded tissue on day 18 after wounding appear in Fig. 5A and fig. S26. The yellow dashed line encircles the wound site. The granulation tissue thickness for the control, untreated, and treated groups are 195 ± 33, 222 ± 52, and 595 ± 50 μm, respectively (*n* = 9; ***P < 0.001; Fig. 5C). Masson’s trichrome staining of the wound on day 30 after wounding identifies epithelial coverage at the center of the wound (Fig. 5B and fig. S27). As this is a validated, established impaired wound healing model (*28*, *34***)**, Masson’s trichrome staining of the wound on day 30 after wounding allows us to identify complete epithelial coverage at the center of the wound for all three groups. The density and the arrangement of collagen were similar in all three groups, meaning that the thin electrode did not disturb the formation of the connective tissue. The epithelial thickness of the treated group (48 ± 4 μm) is almost three times higher than that of the control (15 ± 2 μm) and untreated (16 ± 2 μm) groups (*n* = 12; ***P < 0.001; Fig. 5D). Results of keratin-10 signal and double staining of CD31 and alpha smooth muscle actin (α-SMA) at day 30 after wounding yield information about the maturation and differentiation of the outer and inner layer of the wound (Fig. 5, E and F). The electrostimulated group exhibits a matured spinous layer and strong keratin-10 signal. The keratin-10 fluorescence intensities for the control, untreated, and treated groups are 2.7 ± 0.6, 2.5 ± 0.4, and 9.3 ± 0.7, respectively (*n* = 5; **P < 0.01; Fig. 5J). This group also shows enhanced microvasculature formation (112 ± 11 vessels/mm^2^) compared to the untreated (26 ± 4 vessels/mm^2^) and control groups (29 ± 2 vessels/mm^2^) (*n* = 3; *P < 0.05; Fig. 5K).

### Modulation of inflammatory responses

In chronic wounds, endogenous electric fields are typically absent because of prolonged inflammation (fig. S6A), thereby impairing the healing process. Electrostimulation is known to reduce proinflammatory and stimulate anti-inflammatory responses (*35*, *36*). Experiments to examine these effects involve three groups of the wounded tissue assessed via histology on day 4 after wounding. Immunofluorescence staining for the macrophage cell marker, F4/80, and proinflammatory cytokine, interleukin-6 (IL-6), indicates that electrostimulation reduces inflammatory responses (Fig. 5, G and H). The mean fluorescence intensity associated with F4/80 is 1.6 ± 0.3 for the control group, 1.8 ± 0.5 for the untreated group, and 0.57 ± 0.02 for the treated group (*n* = 6; Fig. 5L). Similarly, fluorescence due to IL-6 is 1.4 ± 0.1, 1.5 ± 0.2, and 0.53 ± 0.1 for the control, untreated, and treated groups, respectively (*n* = 6; Fig. 5M). For the anti-inflammatory cytokine IL-10 (Fig. 5I), the corresponding values are 0.30 ± 0.03, 0.28 ± 0.04, and 1.0 ± 0.1 for the control, untreated, and treated groups, respectively (*n* = 6; *P < 0.05 and **P < 0.01; Fig. 5N). The electrodes do not induce any additional inflammatory responses compared to those of the control group. Electrostimulation thus leads to the transition from the early inflammation stage to the next stage by subduing the proinflammatory and stimulating the anti-inflammatory response.

### Bioresorbable and biocompatible Mo electrodes in vivo

**Fig. 6.**
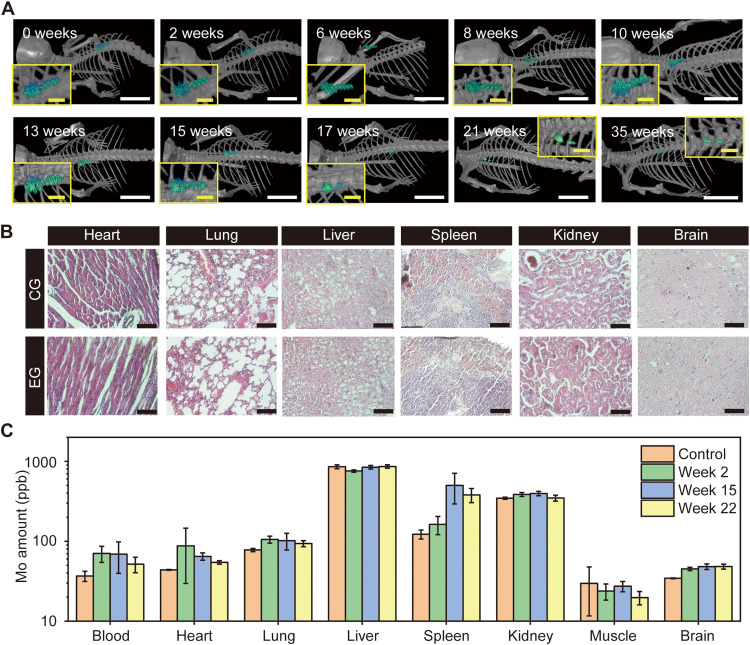
In vivo biodegradation studies. (**A**) Micro-CT images indicate gradual degradation of the device. Scale bars, 1 cm and 2 mm (inset). (**B**) Histological analysis of key organ tissues week 22 after implantation. Scale bars, 100 μm. (**C**) In vivo biodistribution of key elements by ICP-MS (*n* = 4). All data are represented as means ± SD. CG, control group; EG, experimental group; ppb, parts per billion.

Micro–computed tomography (micro-CT) yields high-resolution images for monitoring the bioresorption of the Mo electrode. A small piece of the inner Mo electrode implanted into the mouse maintains its original shape over 13 weeks, meaning that the stimulation and sensing are consistent and predictable throughout the healing period and then gradually resorbs in the body (Fig. 6A). In vivo bioresorption tests demonstrate that the Mo electrode almost disappears after 35 weeks (245 days), which is similar to the estimated lifetime of the Mo electrode calculated by accelerated testing (around 300 days). Histology and biodistribution of Mo associated with bioresorption in a mouse model reveal aspects related to toxicity. Comparison between the control group and experimental group of the histological analysis of key organ tissues (heart, lung, liver, spleen, kidney, and brain; 22 weeks after Mo electrode implantation) indicates no damage to the tissue, no discernible immune response, and no distinguishable Mo flakes/particles (Fig. 6B). Figure 6C shows the concentration of Mo in the blood, heart, lung, liver, spleen, kidney, muscle, and brain tissues explants from mice at 2, 15, and 22 weeks after implantation, measured by ICP-MS. The organs of the control group and those with implanted Mo electrodes reveal no abnormal accumulation of Mo in the organs but a small accumulation during the first 2 weeks’ implantation period. In the blood, heart, lung, and kidney, the Mo concentration gradually decreases and returns to a range similar to the control group after 22 weeks of implantation. Most of the Mo by-products accumulate in the spleen, which easily stores nanoscale particles after week 15 of implantation (*37*). After 22 weeks, the micro-CT images confirm that almost all of the Mo resorbs in the body, and the concentration of Mo in the spleen also begins to decrease. In the brain, the concentration of Mo saturates after 22 weeks of implantation, and we expect them to be decreased at a later time point, as previously reported (*38*).

## DISCUSSION

The results reported here demonstrate that a bioresorbable, wireless, and battery-free electrotherapy system can serve as an effective and unique platform for accelerating and monitoring the processes of wound healing in diabetic small animal models. The electrode designs support levels of electrical conductivity and interface impedances that are necessary for electrotherapy and wound monitoring over several weeks of use in a thin, flexible, and stretchable construct that naturally bioresorbs into the healed tissue to eliminate the need for surgical retrieval. The current measured during the stimulation serves as a parameter related to the healing process, dependent on drying of the wound as a crucial aspect of the healing process. The gradual decrease in current measurement relates directly to progressive healing. Pairing these compliant, bioresorbable electrodes with a compact wireless electronics module and graphical user interface yields a complete system with attributes that not only facilitate animal studies but also potentially treat and monitor chronic wounds in home settings. The placement of one device is expected to be sufficient to treat and monitor chronic wounds without the need for multiple skin care products. Although it is known that a high source of voltage inhibits bacterial growth (*39*), the naturally occurring endogenous current that we are attempting to restore or mimic did not have bactericidal effects (fig. S28). However, we would expect the device to inhibit bacterial growth as Mo oxidizes into molybdenum oxides that have been reported to have bactericidal effects (*40*). This study is a first step in an established rodent model of diabetic impaired wound healing that assesses re-epithelialization and new tissue formation due to the presence of the splints that minimize wound contraction (*28*, *34*). Although this limitation requires caution when interpreting potential results in humans, the rodent model will enable us to investigate and develop additional enhancements such as integrating capabilities for programmed drug delivery, biochemical/biophysical sensing, and closed-loop control of operational parameters, which must also be evaluated in a larger animal model.

## MATERIALS AND METHODS

### Fabrication of a bioresorbable, wireless, and battery-free electrotherapy system

A laser cutting process applied to uniform foils of Mo (15 or 25 μm in thickness; Goodfellow) formed the bioresorbable metal inner and outer electrodes. Schematic diagrams and the board layouts for the wireless platform were designed using Autodesk EAGLE (version 9.6.0). The components included 0201 inch footprint passive elements (capacitors, resistors, and Schottky diodes), seven turn coils for wireless powering (resonant frequency, 13.56 MHz), a microcontroller (ATiny84A), low dropout linear regulators (*V*_out_ = 2.8 and 1.1 V), an NFC (M24LR04E-R), amplifier (OPA330AIYFFR), and a red LED. A silicone elastomer (Silbione-4420) formed an encapsulating structure for the wireless platform. All samples underwent sterilization by ethylene oxide (EtO) gas (Anprolene, AN74i) before in vivo studies.

### Electrical and heating simulations

FEA was implemented on the commercial software COMSOL 5.2a by coupling the Electric Current, Heat Transfer, and Electric Circuit Modules to determine the electric field, current density, current in the electrodes, and the temperature change in the wound and tissue layers of the mouse for a voltage of 1.1 V applied to the electrode.

The stationary form of the partial differential equation for the electric current is

∇⋅J=0 (1)

where **J** is the current defined as **J** = σ**E**. The electric field is given by **E* = −***∇*V*, where σ is the electrical conductivity and *V* is the electrical potential in the electrode.

The heat transfer process is governed by

$$\rho C_{p}\;\cdot \nabla T=\nabla \;\cdot (k\nabla T)+Q_{e}$$ (2)

where ρ is the density, *C*_p_ is the specific heat capacity, *k* is the thermal conductivity, and *T* is the temperature field. The Joule heating effect is introduced by the term *Q*_e_ = **J·E**. The electrode terminals were connected in series to a DC voltage source and resistor *R* = 100 kilohms through the external *I* versus *U* feature that links the physics interfaces.

The Mo electrode and the mouse tissue layers were modeled using four-node tetrahedral elements. A convergence test of the mesh size was performed to ensure accuracy. The total number of elements in the models was approximately ~2 million. The thickness and material properties of the tissue layers used in the simulation are listed in Table 1.

**Table 1.** Electrode and tissue layer properties used in the multiphysics simulation.

Electrode and tissue layers	Thickness (μm)	Density (ρ) (kg/m^3^)	Specific heat capacity (*C_p_*) (J/kg·K)	Thermal conductivity (*k*) (W/m·K)	Relative permittivity (ε)	Electrical conductivity (σ) (S/m)
Molybdenum	15	10,200 (*42*)	250 (*42*)	138 (*42*)	1 (*42*)	2 × 10^7^ (*42*)
Stratum corneum	5 (*43*, *44*)	1090 (*45*)	3350 (*45*)	0.209 (*46*)	5 × 10^2^ (*41*, *47*)	2 × 10^−6^ (*41*, *47*)
Viable epidermis	22 (*43*, *44*)	1090 (*45*)	3350 (*45*)	0.209 (*46*)	5 × 10^6^ (*41*, *47*)	0.026 (*41*, *47*)
Dermis	280 (*43*, *44*)	1090 (*45*)	3350 (*48*)	0.322 (*46*)	5 × 10^9^ (*41*, *47*)	0.222 (*41*, *47*)
Subcutaneous fat (adipose tissue + panniculus carnosus muscle)	300 (*48*, *49*)	911 (*50*)	3660 (*51*)	0.21 (*46*)	5 × 10^7^ (*41*, *47*)	0.08 (*41*, *47*)
Muscle	2000 (*52*)	1060 (*53*)	3730 (*54*)	0.530 (*55*)	2 × 10^7^ (*41*, *47*)	0.25 (*41*, *47*)

### Electromagnetic simulation

FEA was conducted for electromagnetic simulations to study the magnetic field distribution around the transmitting antenna. The simulations were performed using the commercial software Ansys HFSS, where tetrahedron elements were used in the solution with adaptive meshing convergence. An adaptive mesh convergence condition and a spherical radiation boundary (1000 mm in radius) were adopted to ensure computational accuracy. The electromagnetic parameters in the material library of Ansys HFSS were used in the simulation.

### Mechanical simulation

3D FEA was used to design the Mo electrode and to predict the strain under stretching, bending, and twisting. Eight-node 3D solid elements and four-node shell elements were used for the tissue and Mo electrode, respectively. Convergence of mesh sizes was tested to ensure computational accuracy using the commercial software ABAQUS. In the FEA model, the Mooney-Rivlin strain energy potential model was used for the tissue (elastic modulus *E*_tissue_ = 130 kPa and Poisson’s ratio ν_tissue_ = 0.49) displaying hyperelastic material behavior, where the relevant materials parameters include C10 = 0.0174 MPa, C01 = 0.0044 MPa, and D1 = 0.923 MPa^−1^. Molybdenum was modeled with ideal elastoplastic behavior, where the elastic modulus, Poisson’s ratio, and elastic strain limit are *E*_Mo_ = 315 GPa, ν_Mo_ = 0.29, and ɛ_Mo_ = 0.35%, respectively.

### The diabetic wound healing model

All in vivo studies were approved by the Institutional Animal Care and Use Committee at Northwestern University (protocols IS00000373 and IS00018748). Diabetic (db/db) mice (BKS.Cg-m +/+ Leprdb, #000642; homozygous for Leprdb) were purchased from the Jackson laboratory (Bar Harbor, ME, USA). A splinted excisional wound model in db/db mice (BKS.Cg-Dock7m +/+ Leprdb/J Homozygous for Leprdb) was used, as previously described (*41*). To prevent skin contraction, paired sterilized doughnut-shaped acrylate splints (inner diameter, 10 mm; outer diameter, 12 mm; 3M, St. Paul, MN) were attached to the left and right dorsal sides of the mouse with Vetbond (3M) and interrupted 6-0 nylon sutures (Ethicon, Cincinnati, OH) after depilation. A 6-mm circular, the full-thickness wound was made in the center of each splinted area. For the groups with devices, each device was laminated with the inner electrode on the center of the wound and the outer electrode along the edge of the wound. A transparent sterile occlusive dressing, Tegaderm (3M), was then placed over the wound and the splint. The mouse was monitored every day, and digital images of the wound area were taken every 3 days and quantified by three blinded observers using ImageJ by normalizing the wound area to the known splint area at each time point. The treated group had Mo electrodes with DC electrostimulation for 30 min every day until full wound closure; the untreated group had Mo electrodes without electrostimulation. The control groups did not have Mo electrodes. In addition, for the treated group, the endogenous current was monitored for 5 min daily until there was no signal.

### Tissue processing and immunofluorescence staining

For tissue processing and histology, upon 30, 18, or 4 days after creating the wound, animals were euthanized, and the regenerated wound tissue was excised with a 10-mm biopsy punch (Acuderm, Fort Lauderdale, FL), fixed using 4% paraformaldehyde, and embedded by paraffin. The tissues were then sectioned and stained with H&E to measure granulation tissue thickness. The tissues were also stained with Masson’s trichrome to measure the epithelial thickness. The granulation tissue and epithelial thickness were quantified by measuring the thickness at five evenly spaced locations from the center of the wound for each animal using ImageJ. The average of the measurements obtained from each wound was calculated and compared among treatment groups.

Moreover, the tissue sections were stained for keratin-10, α-SMA, CD31, F4/80, IL-6, or IL-10 (Santa Cruz Biotechnology, Dallas, TX). The secondary antibodies were either conjugated to Alexa Fluor 488 or Alexa Fluor 555 (Invitrogen, Carlsbad, CA). Controls consisted of samples stained with the secondary antibody without incubation with a primary antibody. The development of neovascularization and changes in inflammation were quantified using ImageJ.

### Micro-CT and fundamental organ analysis

Mice were anesthetized with isoflurane and placed on the heated bed of the micro-CT system. Images were acquired with a preclinical micro–positron emission tomography scanner/CT imager, nanoScan scanner (Mediso USA, Arlington, VA). Data were acquired with “medium” magnification, <60-μm focal spot, 1 × 1 binning, with 720 projection views over a full circle, with a 300-ms exposure time. Images were acquired using 70 kVp. The projection data were reconstructed with a voxel size of 34 μm and using filtered (Butterworth filter) backprojection software from Mediso. The reconstructed data were visualized and segmented in Amira 2020.2 (FEI, Houston, TX).

For the histological analysis of essential organs (heart, lung, liver, spleen, kidney, and brain), mice were euthanized after 2, 15, and 22 weeks of implantation. Organs were harvested and fixed using 4% paraformaldehyde and then embedded with paraffin. The organs were then sectioned and stained with H&E for qualitative analysis.

### ICP-MS study

The process for quantifying the amount of Mo in targeted tissues used ICP-MS applied to acid samples digested by immersion trace–grade nitric acid (HNO_3_; >69%; Thermo Fisher Scientific, Waltham, MA, USA) and trace grade hydrogen peroxide (H_2_O_2_; 30.0 to 32.0%; GFS Chemicals, Columbus, OH, USA) and then heating to 65°C for at least 3 hours. The addition of ultrapure H_2_O (18.2 megohm∙cm) yielded a final solution of 5.0% nitric acid. Specific volumes of nitric acid, hydrogen peroxide, and final solutions are given in Table 2 according to tissue type.

**Table 2.** Volumes of nitric acid, hydrogen peroxide, and final solutions according to tissue type.

Tissue type	Approximate weight of tissue used (g)	Volume HNO_3_ (ml)	Volume H_2_O_2_ (ml)	Final volume (ml)
Whole blood	0.2	0.25	0.25	5
Liver	1–1.5	2	0.5	40
All others	0.1–0.5	0.5	0.125	10

Quantitative standards used a Mo standard solution (1000 μg/ml; Inorganic Ventures, Christiansburg, VA, USA) to create Mo at 100 ng/g in 5.0% nitric acid (v/v) in a total sample volume of 50 ml. A solution of 5.0% nitric acid (v/v) was used as the calibration blank.

ICP-MS used a computer-controlled (Qtegra software) Thermo iCAP Q ICP-MS (Thermo Fisher Scientific, Waltham, MA, USA) operating in KED mode and equipped with an ESI SC-2DX prepFAST autosampler (Omaha, NE, USA). The internal standard was added inline using the prepFAST system and consisted of a mixed element solution at 1 ng/ml containing Bi, In, 6Li, Sc, Tb, and Y (IV-ICPMS-71D from Inorganic Ventures). Online dilution was also performed by the prepFAST system and used to generate a calibration curve consisting of 100, 50, 20, 10, 1, 0.5–part per billion Mo. Each sample was acquired using one survey run (10 sweeps) and three main (peak jumping) runs (40 sweeps). The isotopes selected for analysis were 92,95,96Mo and 90Zr, 101Ru (chosen to perform interference corrections), and 89Y, 115In (chosen as internal standards for data interpolation and machine stability). Instrument performance was optimized daily through autotuning, followed by verification via a performance report (passing manufacturer’s specifications).

### In vitro biocompatibility test

For the in vitro viability assay, a mouse fibroblast cell line(L929) was purchased from the American Type Culture Collection (ATCC; CCL-1) along with their media (ATCC, 30-2003). Cells were maintained and cultured in T-25 flasks according to the manufacturer’s protocols. After 10,000 cells were seeded onto the 24-well plate, EtO gas–sterilized electrodes were placed on top. After maintaining them for 96 hours, resazurin assay and live and dead staining were performed as the manufacturer’s protocol. Resazurin salt was purchased from Sigma-Aldrich (R7017), and the LIVE/DEAD Viability/Cytotoxicity Kit (L3224) was purchased from Invitrogen. Both fluorescence measurement and imaging were done with Cytation5 (BioTek).

### Keratinocyte migration assay

Primary human keratinocytes (KR-F) and their growth medium (KM-2) were purchased from Zen-Bio for the scratch assay. Keratinocytes were maintained according to the manufacturer’s protocols, and cells were used for migration assays after the first or second subculture. To electrostimulate cells, the electrodes were bonded onto a PDMS substrate, and the electrodes were connected to a function generator (SDG1025, Siglent). For fabrication of these components, PDMS (SYLGARD 184, Dow Corning) base was mixed with a curing agent at a weight ratio of 10:1 (base:curing agent) and poured onto a petri dish. After degassing, PDMS was semicured in an oven maintained at 65°C for 15 min. The trimmed Mo electrodes were placed onto semicured PDMS and then fully cured. The electrode with PDMS substrate was then connected to the printed circuit board via anisotropic conductive film (Elform). After the device was EtO gas sterilized and glued to the surface of a six-well plate, the cell culture surface on the device was coated with fibronectin solution (0.1 mg/ml in PBS) (Corning, 356008) for 2 hours of incubation in a cell culture incubator at 37°C and 5% CO_2_. Next, the surface was washed with PBS and filled with culture medium before seeding of human keratinocytes at a concentration of 1 × 10^6^ cells/ml. After an hour of incubation to ensure that the cells settle onto the surface, the well was filled with a culture medium. After 24 hours, scratches were made using a 200-μl pipette tip. Using a function generator, 200 mV/mm was applied for 24 hours. Quantitative analysis was performed using ImageJ.

## Notes

View/request a protocol for this paper from *Bio-protocol*.
