# Supplementary material for: Bioresorbable, wireless, and battery-free system for electrotherapy and impedance sensing at wound sites
Source: Sci Adv. 2023 Feb 22;9(8):eade4687. doi: 10.1126/sciadv.ade4687 (PMC9946359; doi:10.1126/sciadv.ade4687)
Supplement: Supplementary file 1 — Figs. S1 to S28 [file sciadv.ade4687_sm.pdf]

Science Advances
AAAS

Supplementary Materials for

Bioresorbable, wireless, and battery-free system for electrotherapy and impedance sensing at wound sites

Joseph W. Song *et al.*

Corresponding author: Guillermo A. Ameer, g-ameer@northwestern.edu;
John A. Rogers, jrogers@northwestern.edu

Sci. Adv. **9**, eade4687 (2023)
DOI: 10.1126/sciadv.ade4687

This PDF file includes:

Figs. S1 to S28

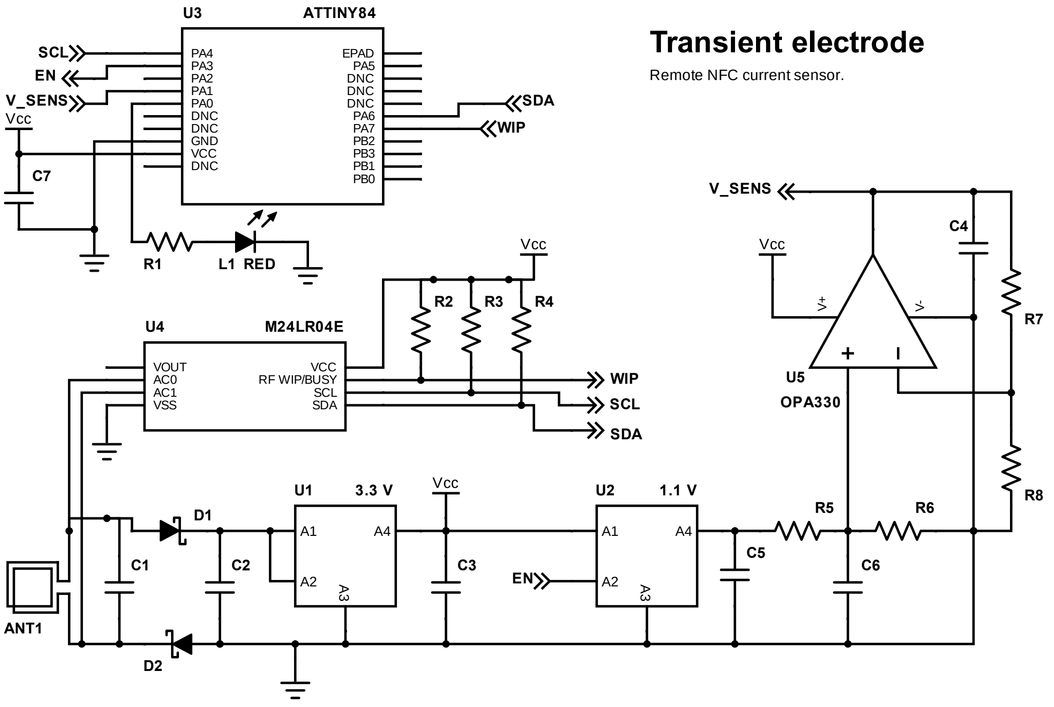

Fig. S1. Circuit diagram for the wireless platform.

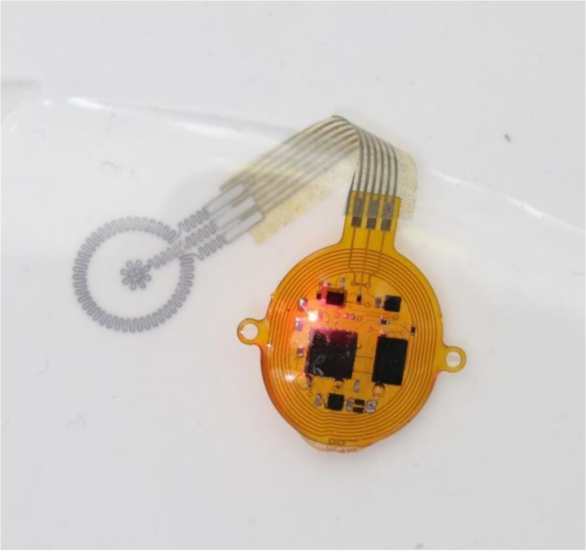

Fig. S2. Picture of a wireless, battery-free electrotherapy system with bioresorbable stimulation electrodes (BES).

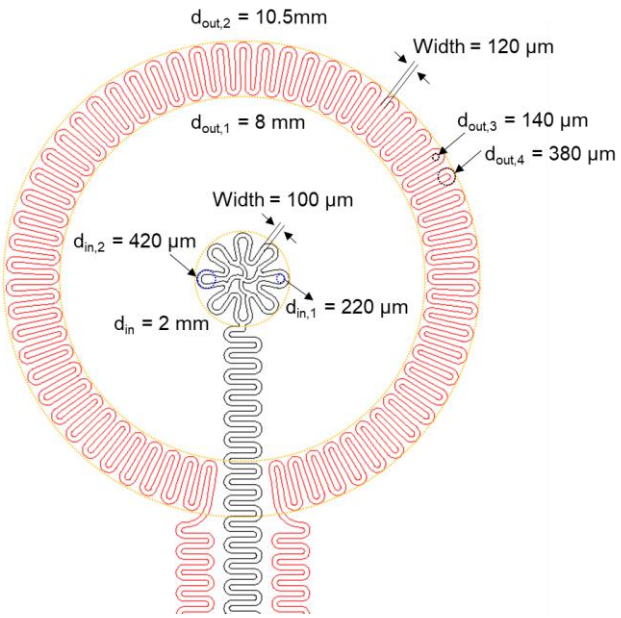

Fig. S3. Design of Mo electrodes with filamentary serpentine traces.

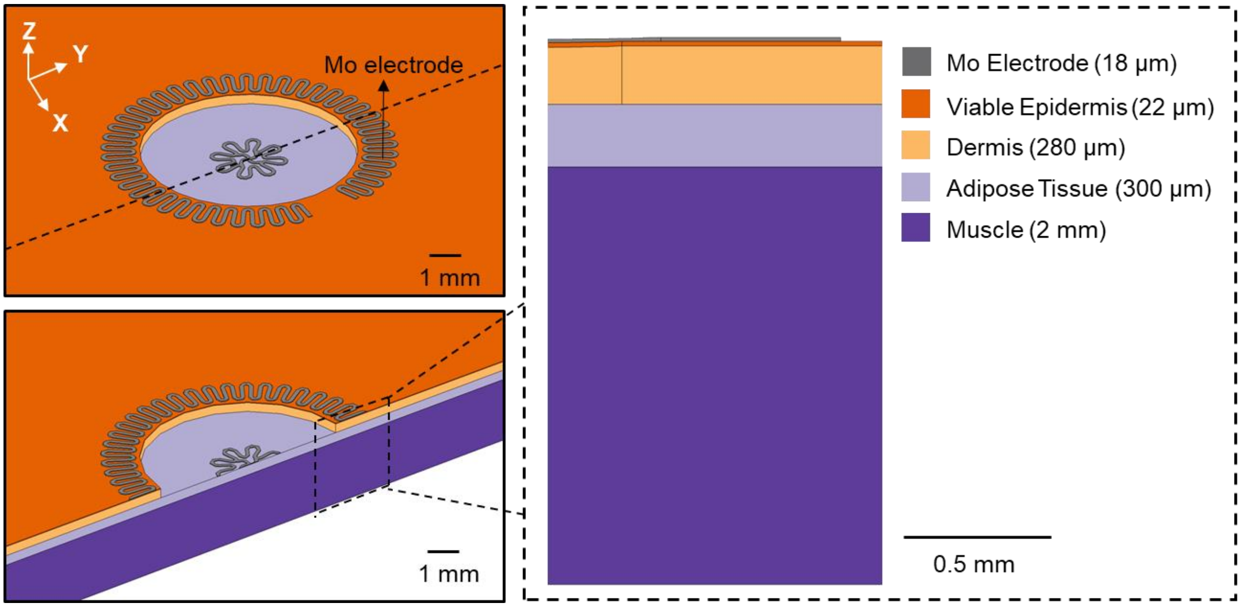

Fig. S4. 3D schematic diagram of the electrode geometries and tissue layer stacking used in the simulations.

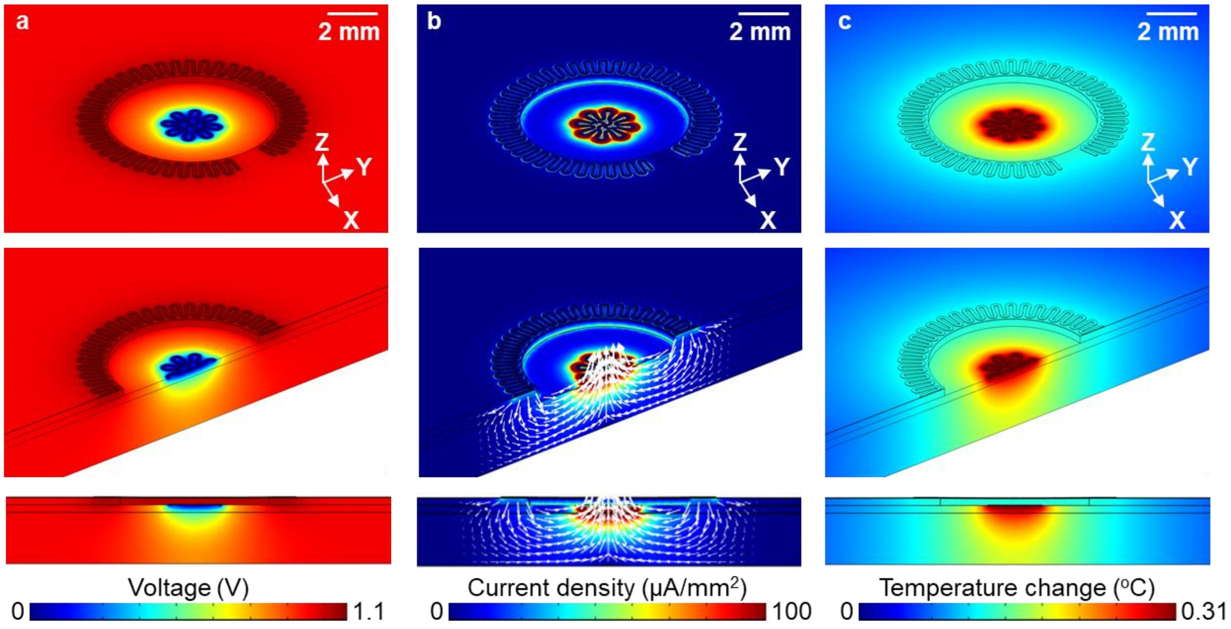

Fig. S5. Electrical finite element analysis of the stimulator.

Simulated (**a**) voltage, (**b**) current density, and (**c**) temperature change in the tissue layers during operation of the stimulator.

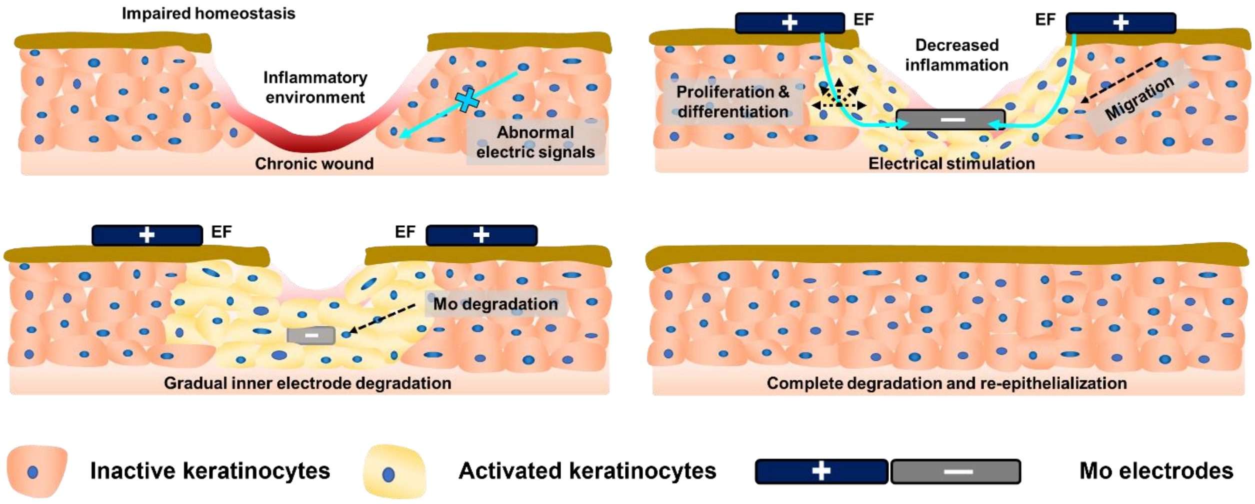

Fig. S6. Healing mechanism of a chronic wound by introducing electrostimulation.

(**a**) Healing of a chronic wound can be frustrated due to prolonged inflammation and impaired homeostasis, which causes abnormal electrical signals. (**b**) The device creates an electric field to activate migration of keratinocytes. (**c**) The inner electrode gradually disappears as it is surrounded by newly generated tissue. (**d**) Complete bioresorption after re-epithelialization and maturation.

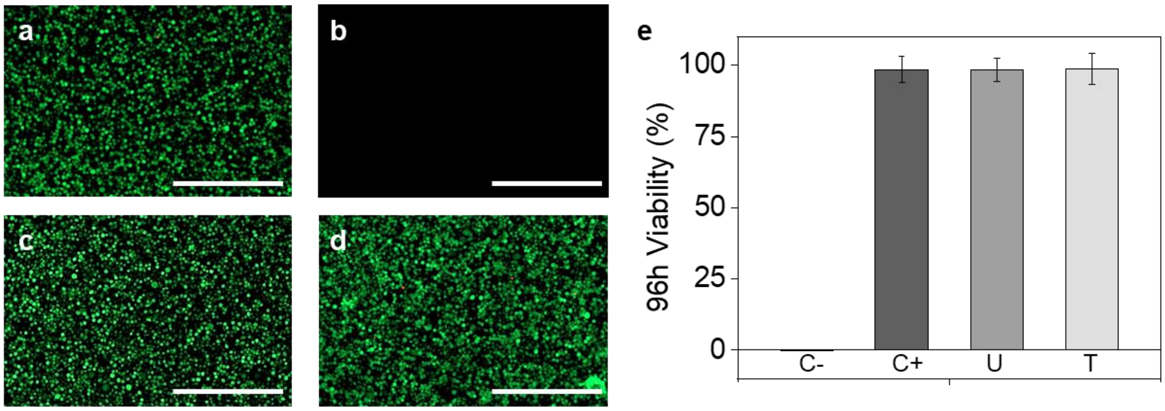

Fig. S7. The Biocompatibility of bioresorbable electrodes.

Tests of the biocompatibility of Mo electrodes in live/dead staining assays of healthy mouse fibroblasts (L929) after 96 hours of culture; (**a**) on tissue culture polystyrene (TCPS) as the positive control; (**b**) DPBS without L929 as the negative control; (**c**) with the Mo electrode without stimulation as the untreated group; (**d**) with the Mo electrode with stimulation as the treated group. scale bar = 500 µm. (**e**) Normalized *in vitro* assay data. n=3 independent samples. All data are represented as mean ± SD.

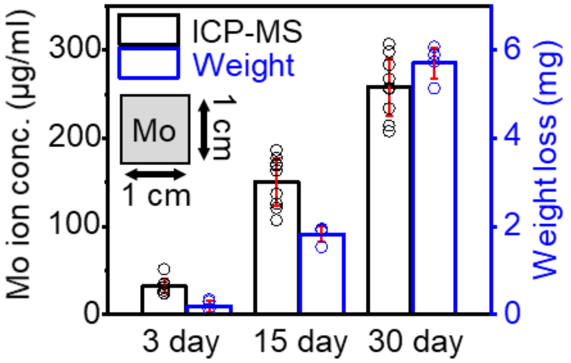

Fig. S8. Dissolution study of Mo electrodes at physiological condition.

Time-dependent concentration of Mo ions in aqueous solution from dissolution of Mo, measured by ICP-MS (n=9, individual samples) and weight loss from 1 cm^2 Mo samples (n=4, individual samples). All data are represented as mean ± SD.

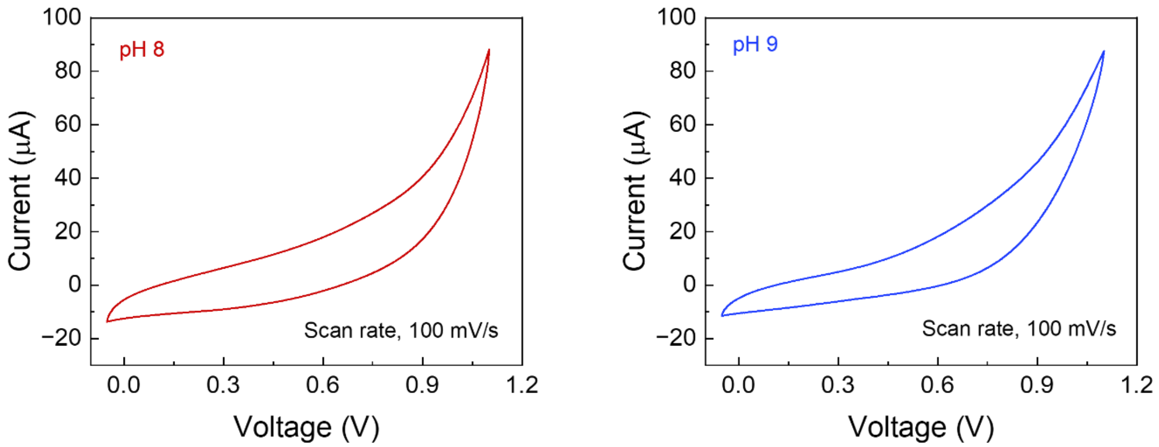

Fig. S9. Cyclic voltammogram of a pair of Mo electrodes as an electrical stimulator during immersion in pH 9.0 and 8.0 buffer at room temperature

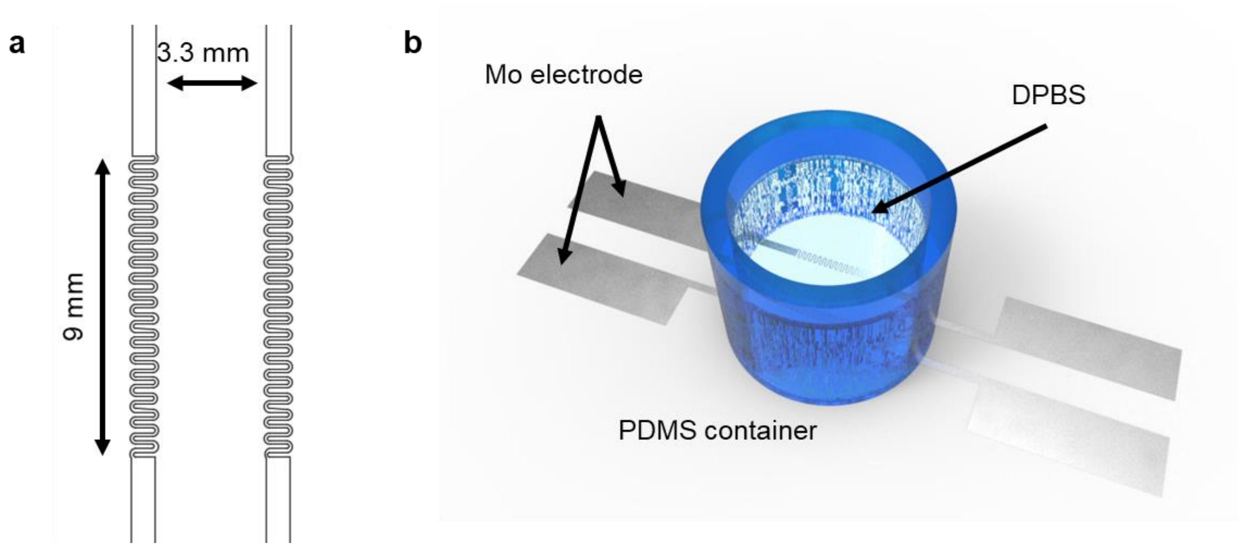

Fig. S10. Experimental conditions of the bioresorbable test.

(**a**) Design of Mo electrodes with serpentine stretchable traces for tests of electrical degradation. (**b**) Schematic illustration of the test environment without a polyimide (PI) top cover film.

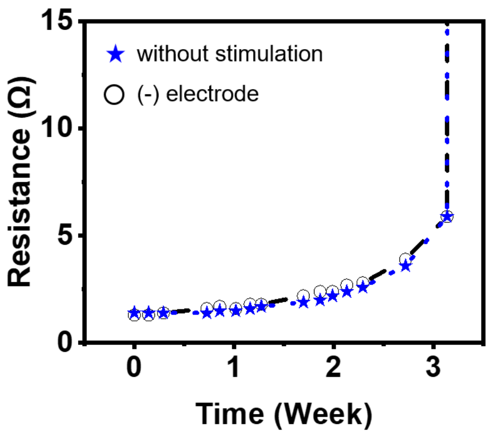

Fig. S11. Electrical properties of the bioresorbable electrode at physiological conditions.

Changes in resistance of a Mo cathode (-) (blue) with an applied voltage of 1.1 V for 30 min/day and a Mo electrode without applied bias (black) during immersion in DPBS (pH 7.4) at 37 °C.

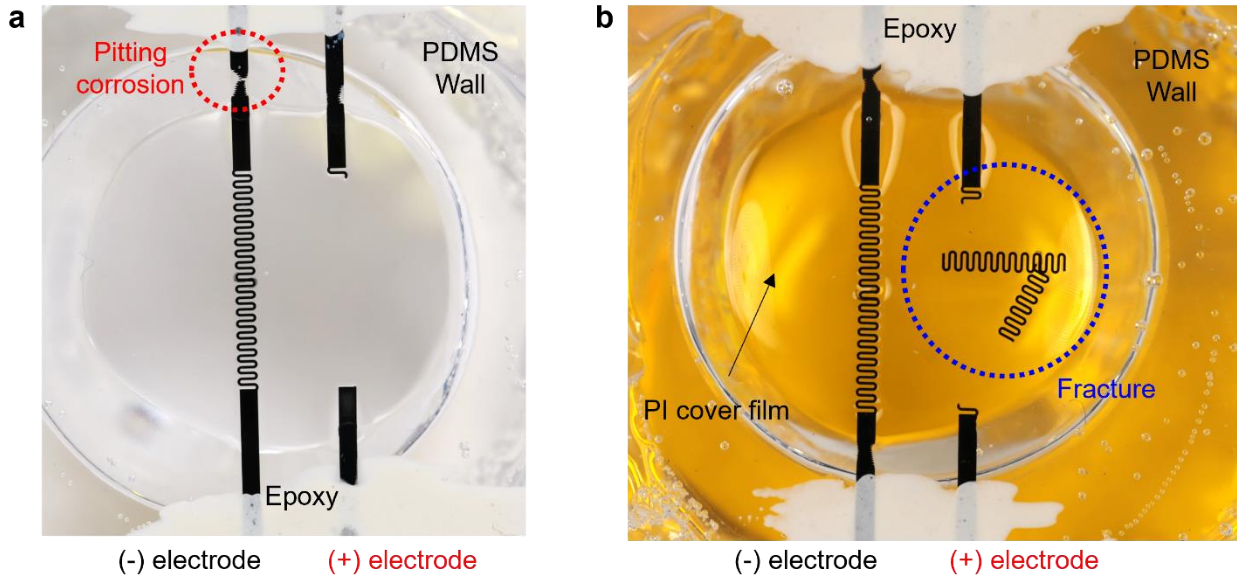

Fig. S12. Two different cases of corrosion behavior of the bioresorbable electrode at physiological conditions.

Pictures of (**a**) pitting corrosion and (**b**) fracture of a dissolved Mo electrode during immersion in DPBS (pH 7.4) at 37 °C for 4 weeks.

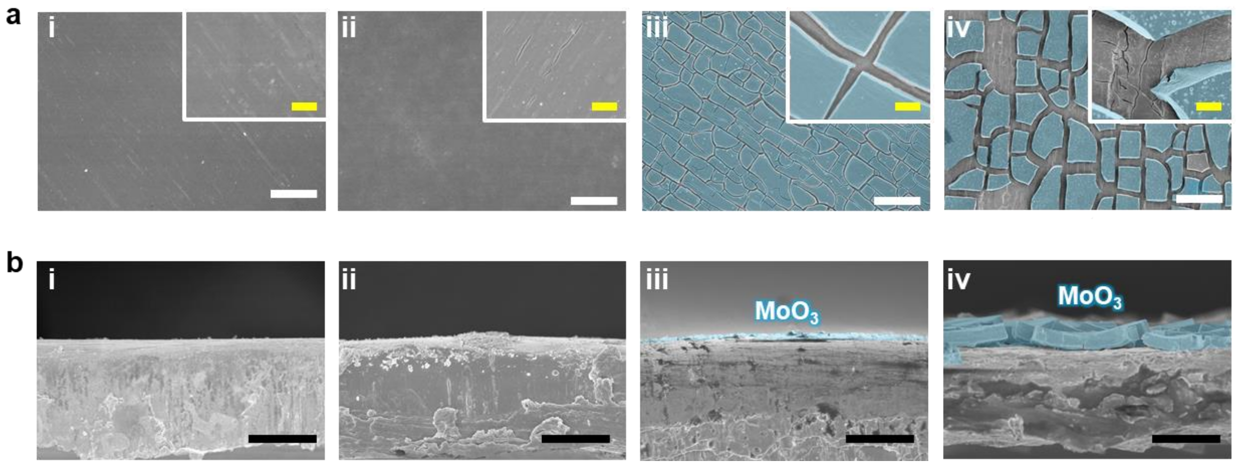

Fig. S13. Time-dependent oxidation of Mo foil at physiological conditions.

(**a**) Surface and (**b**) cross-sectional colorized scanning electron microscope (SEM) images of a Mo foil immersed in DPBS (pH 7.4) at 37 °C after (i) day 0, (ii) day 3, (iii) day 15, and (iv) day 30. The blue color highlights the formation of Mo oxide on the surface. white scale bar = 20 μm, yellow scale bar = 2 μm, black scale bar = 10 μm.

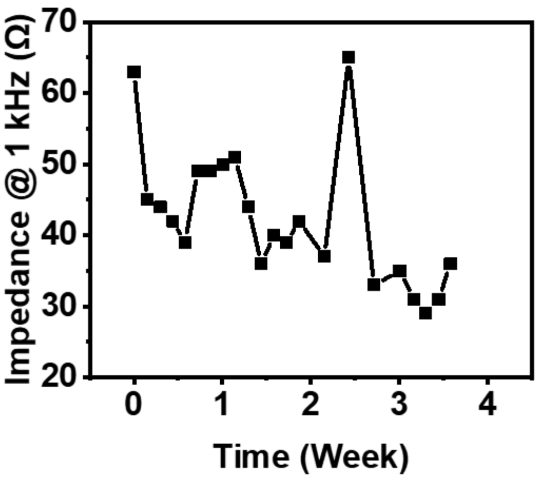

Fig. S14. Changes in impedance at 1 kHz of Mo electrodes for stimulation at 1.1 V for 30 min/day during immersion in DPBS (pH 7.4) at 37 °C.

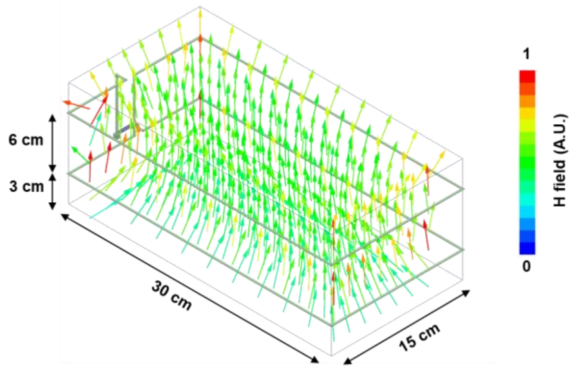

Fig. S15. Magnetic field distribution in the cage, where the arrows indicate the direction and the colors indicate magnitude.

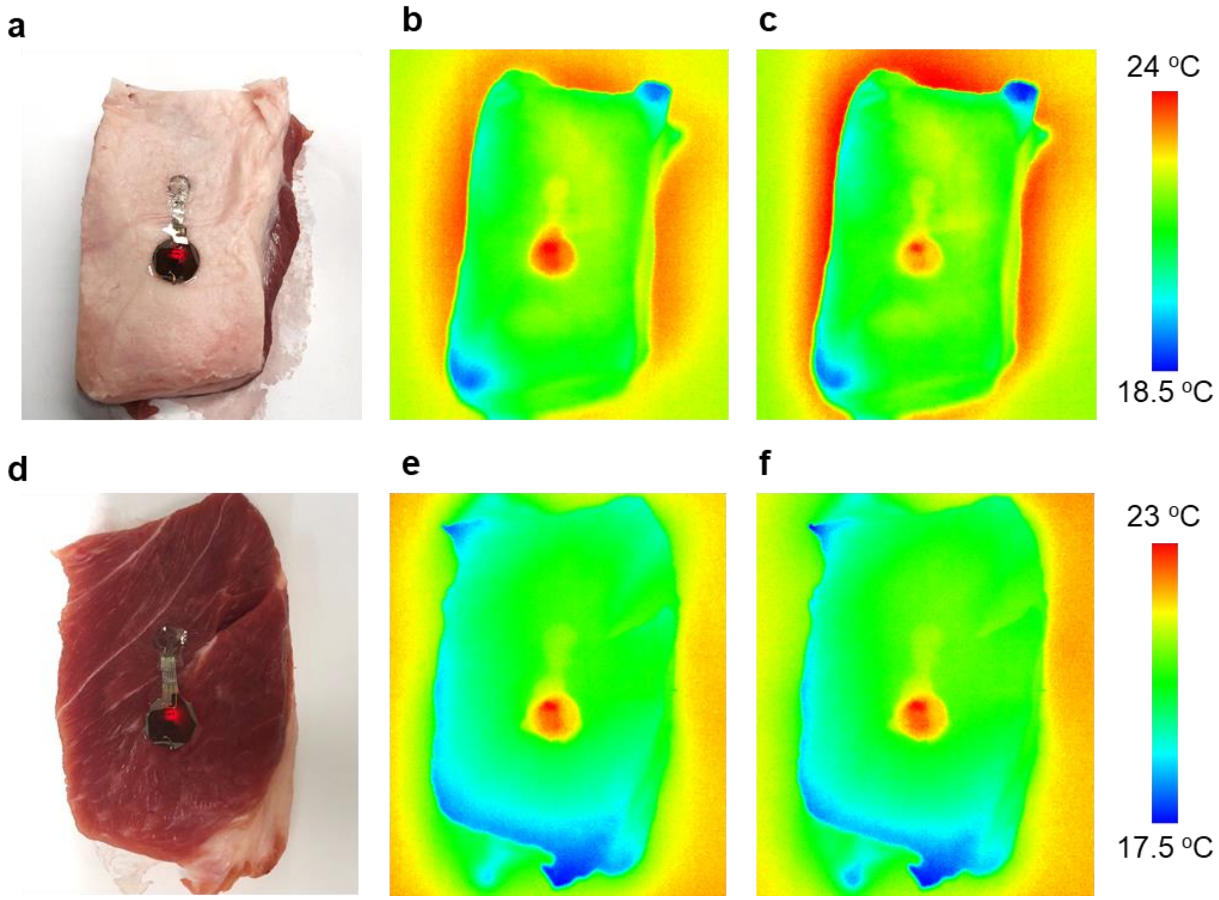

Fig. S16. Thermal stability test of the BES on fat and muscle tissues.

(**a**) Photograph of a device on fat tissue. Infrared image of the device and fat tissue during operation at (**b**) 0 s, and after (**c**) 300 s. (**d**) Photograph of the device on muscle tissue. Infrared image of the device and muscle tissue during operation at (**e**) 0 s, and after (**f**) 300 s.

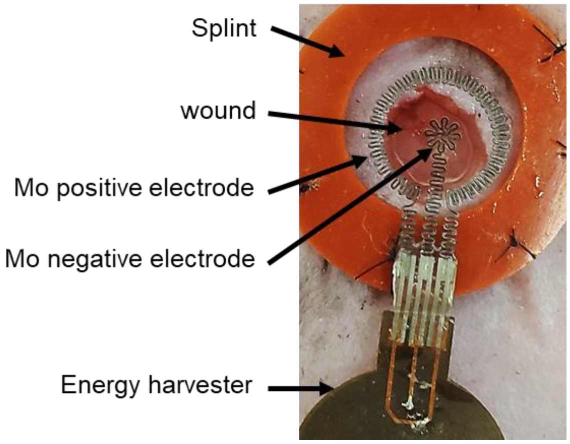

Fig. S17. Photograph of the BES on an excisional wound.

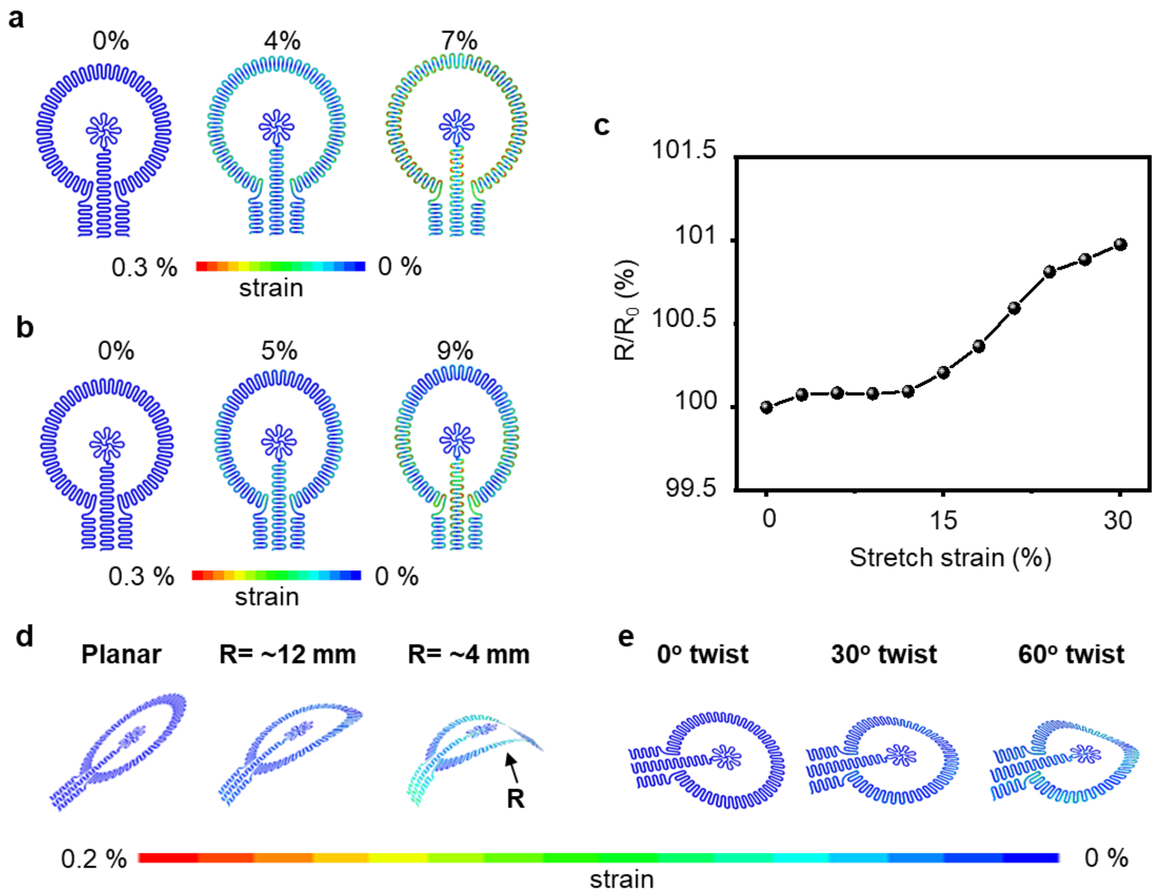

Fig. S18. Mechanical Finite element analysis of the bioresorbable electrodes and electrical properties under different strain conditions.

Finite element analysis (FEA) results for the stimulation electrodes under (**a**) 0%, 4%, and 7% biaxial stretching and (**b**) 0%, 5%, and 9% uniaxial stretching. The color in **a**-**b** represent the equivalent strain. **c**, Changes in resistance of the Mo electrode under different stretching conditions. FEA results of (**d**) bending and (**e**) twisting of Mo electrodes under different conditions. The color represents the equivalent strain.

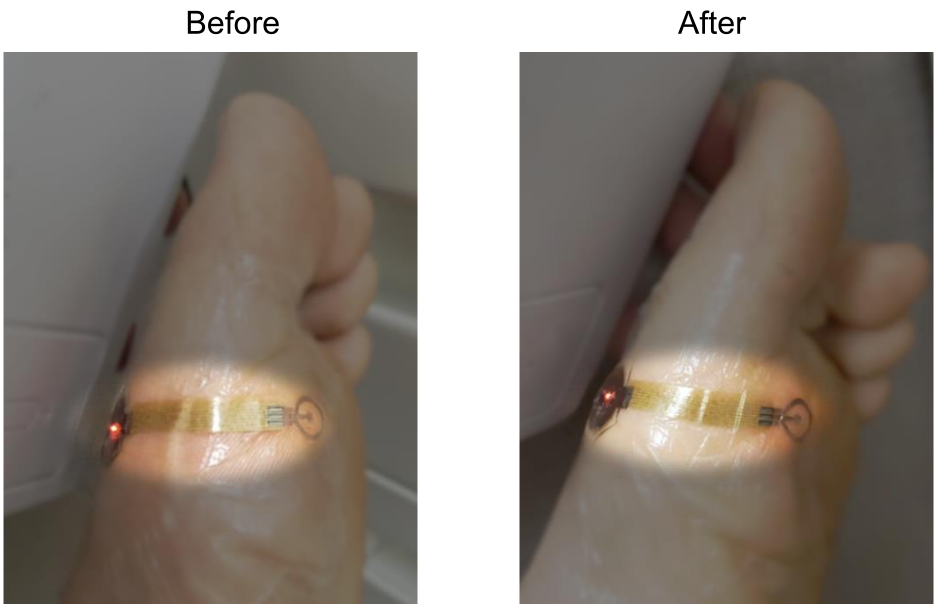

Fig. S19. The device performance was stable after 30 minutes of walking and 30 minutes of jogging when fixed to the base of the foot, fixed with a protective dressing, and covered with a sock and a running shoe.

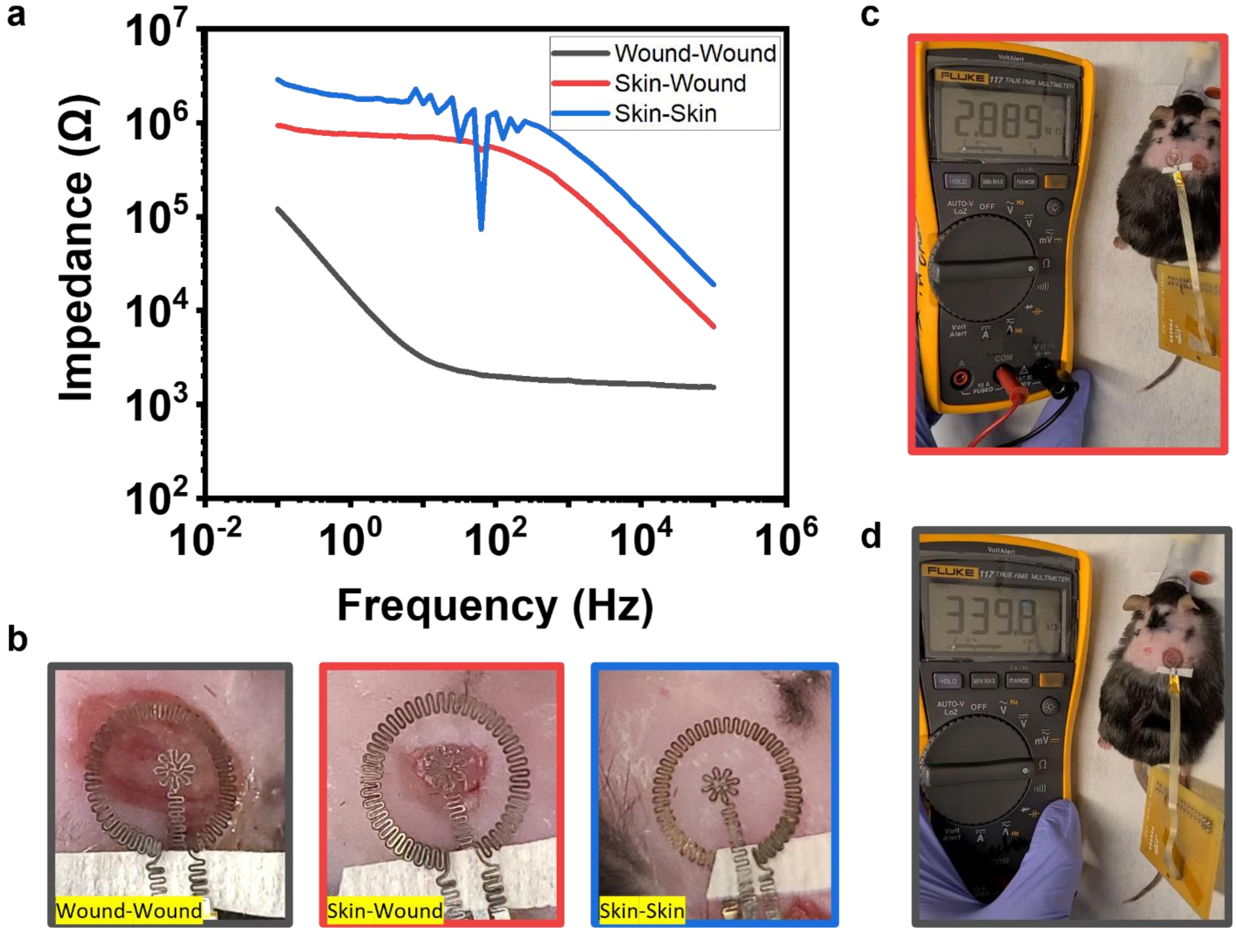

Fig. S20. *In vivo* impedance analysis of the bioresorbable electrodes.

(**a**) Frequency-dependent impedance between the Mo electrodes for both positive and negative electrodes on an excisional wound (black line), for only the negative electrode on an excisional wound (red line), and for both electrodes on the skin (blue line), and (**b**) corresponding experimental images. (**c**) The resistance between the Mo electrodes is 2.889 MΩ when only the negative electrode is on an excisional wound. (**d**) The resistance of between the Mo electrodes is 339.8 kΩ when both Mo electrodes are on an excisional wound.

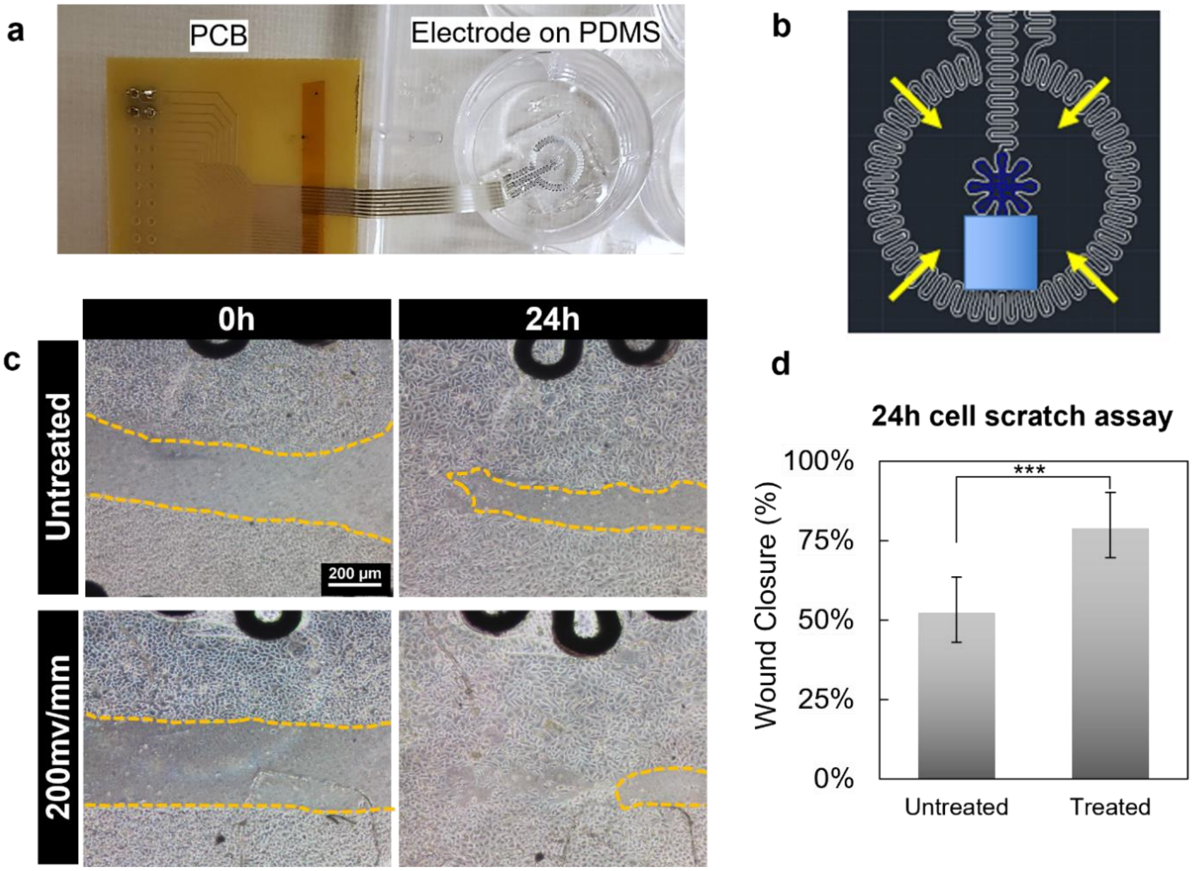

Fig. S21. The study of primary human keratinocytes migration.

Primary human keratinocytes migration was accelerated by electrostimulation. (**a**) In vitro electrotherapy system. (**b**) Area where the studies were performed (**c**) pictures of scratch assay. (**d**) quantification of migration (n=4). All data are represented as mean ± SD.

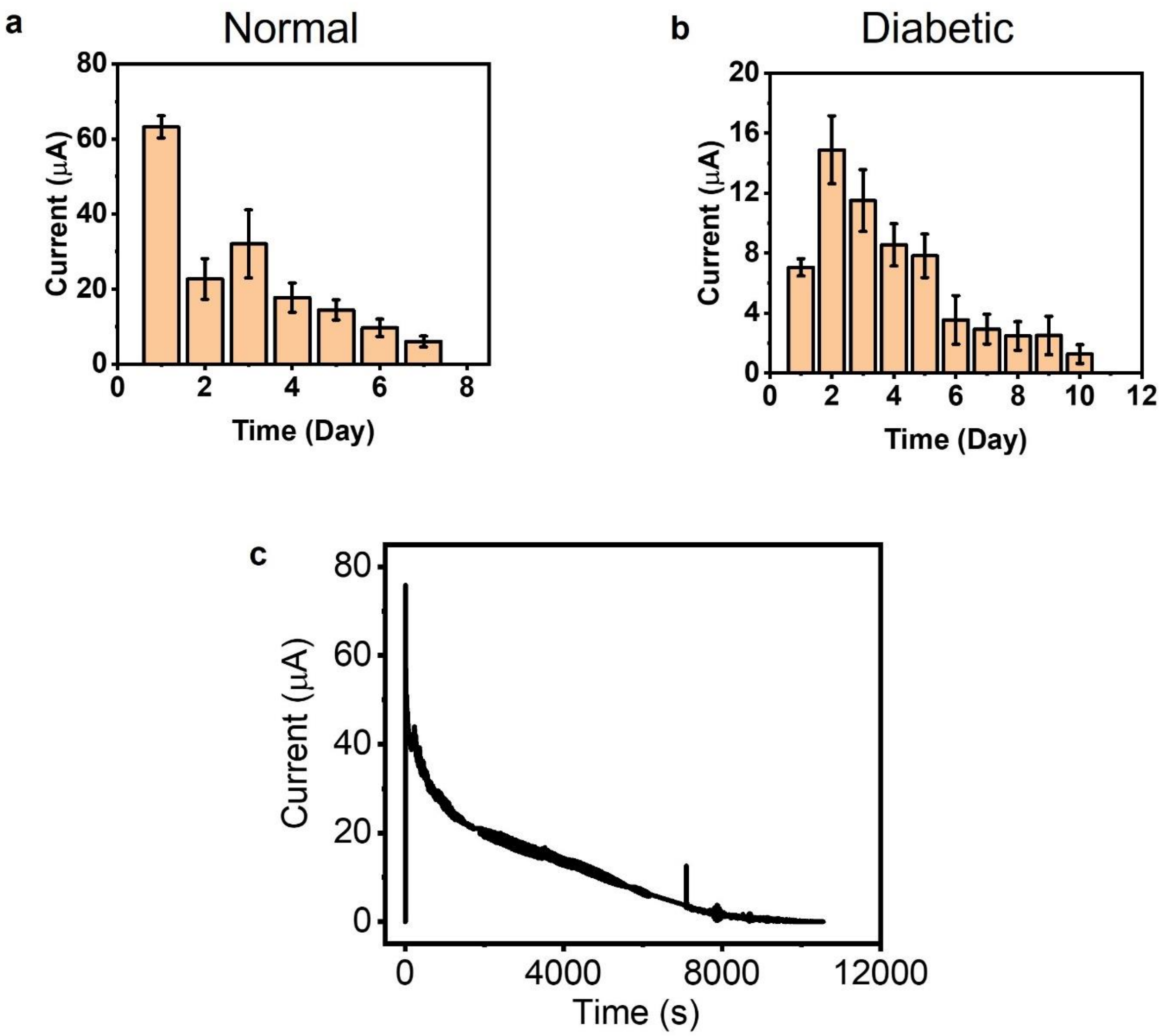

Fig. S22. ***In vivo*** **study of impedance changes during the wound healing process.**
(**a**) Current sensor data on normal mice. (**b**) Current sensor data on diabetic mice. (**c**) The current sensor data on hydrogel until the complete dehydration. All data are represented as mean ± SD.

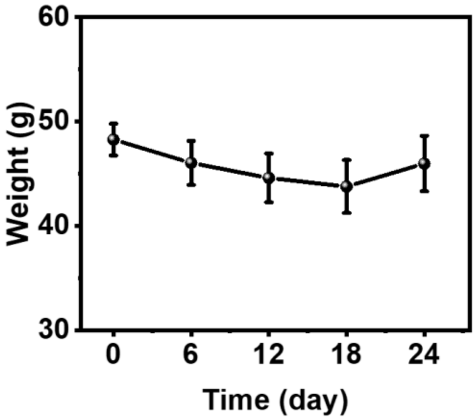

Fig. S23. Time-dependent weight changes of the diabetic mouse during electrostimulation (n=10, individual subjects). All data are represented as mean ± SD.

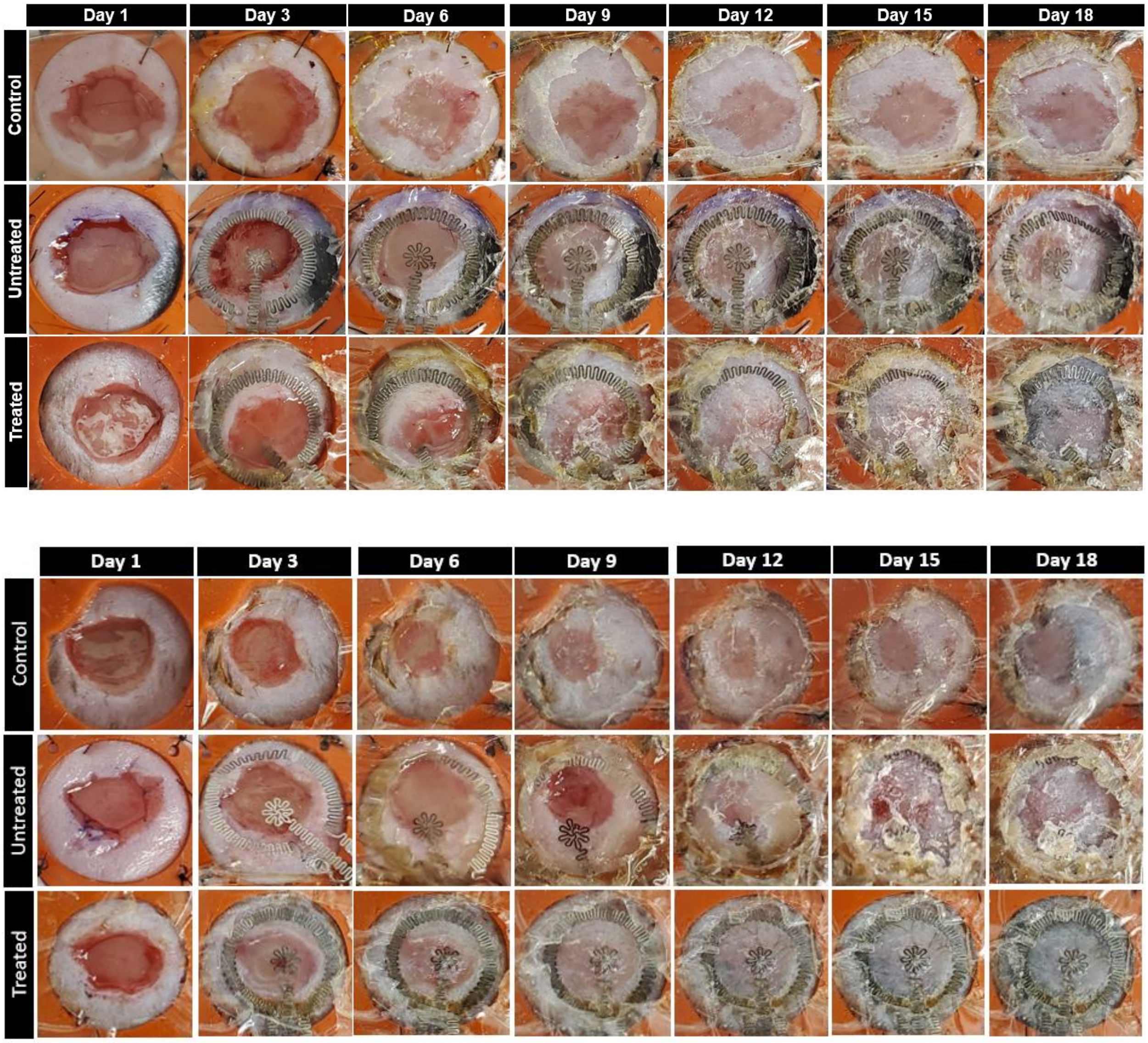

Fig. S24. Two sets of wound closure raw images. The inner diameter of splint is 10mm.

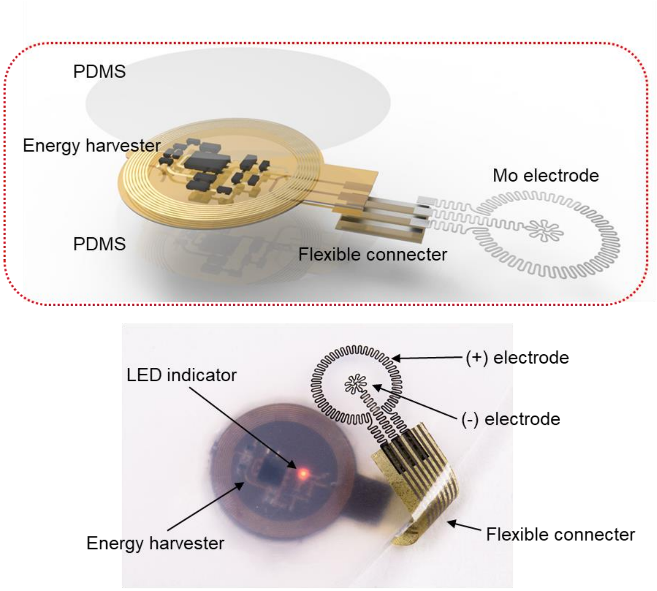

Fig. S25. Schematic and optical image of the simplified transient wound healing system.

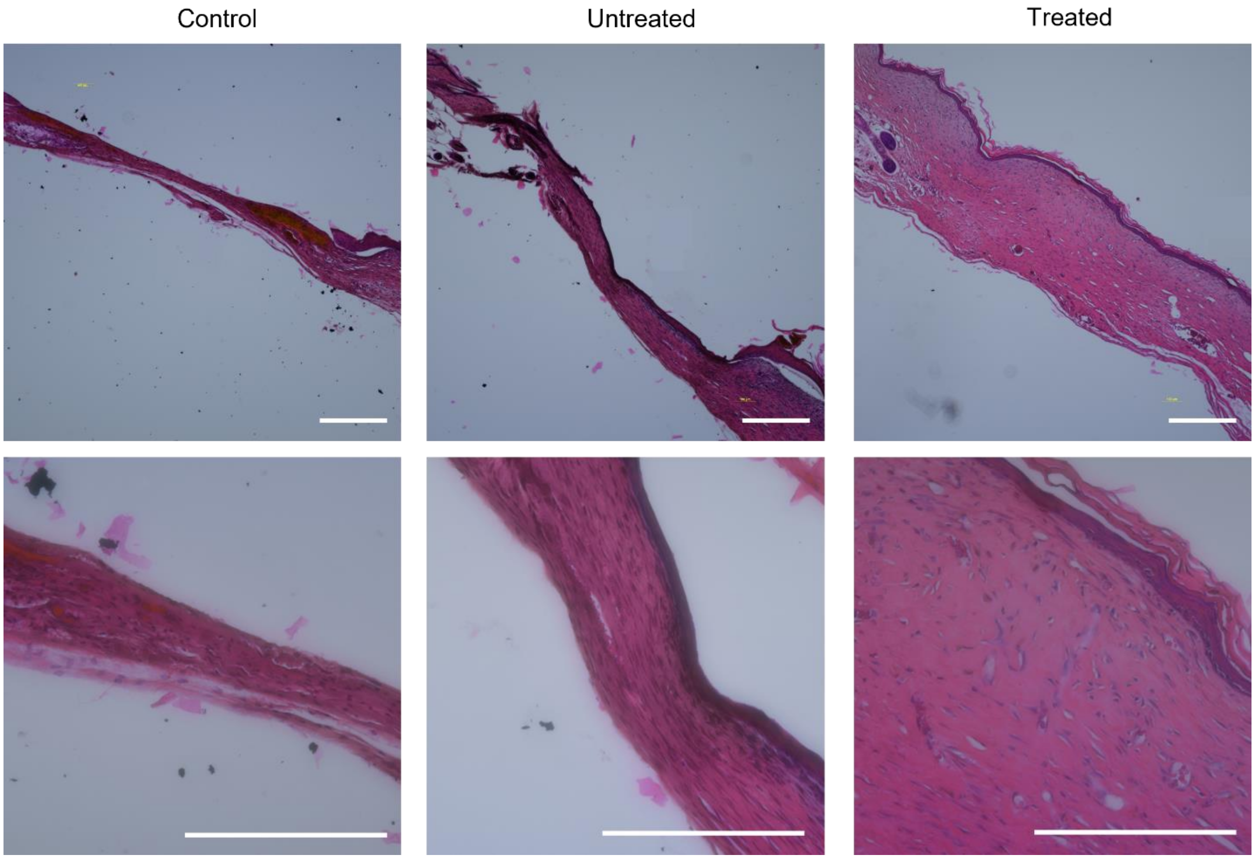

Fig. S26. Magnified digital images of H&E stained tissue at day 18 post wounding (scale bar= 300μm)

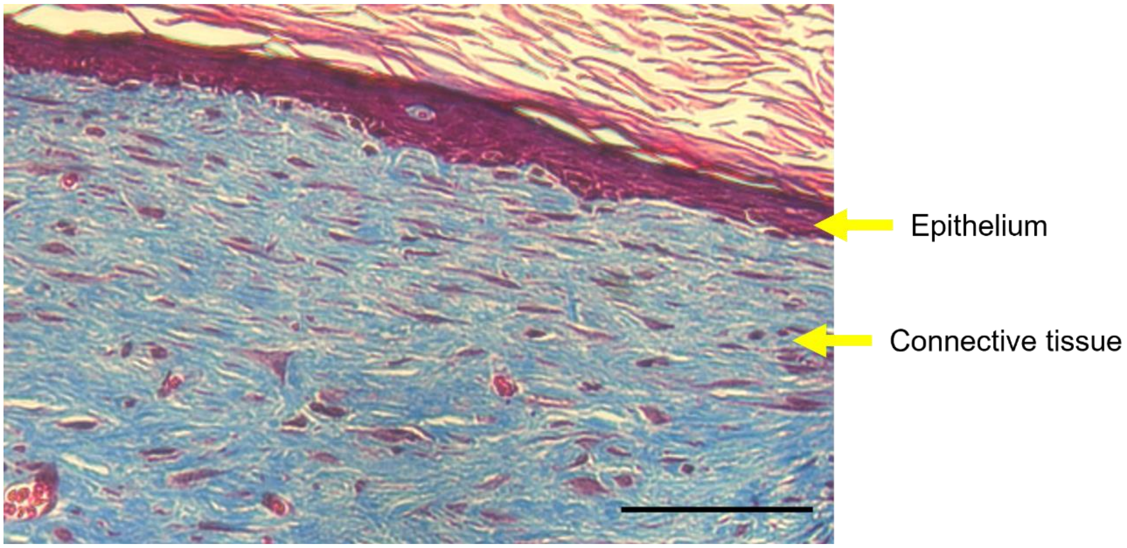

Fig. S27. Masson's trichrome staining with borderline (scale bar= 100μm)

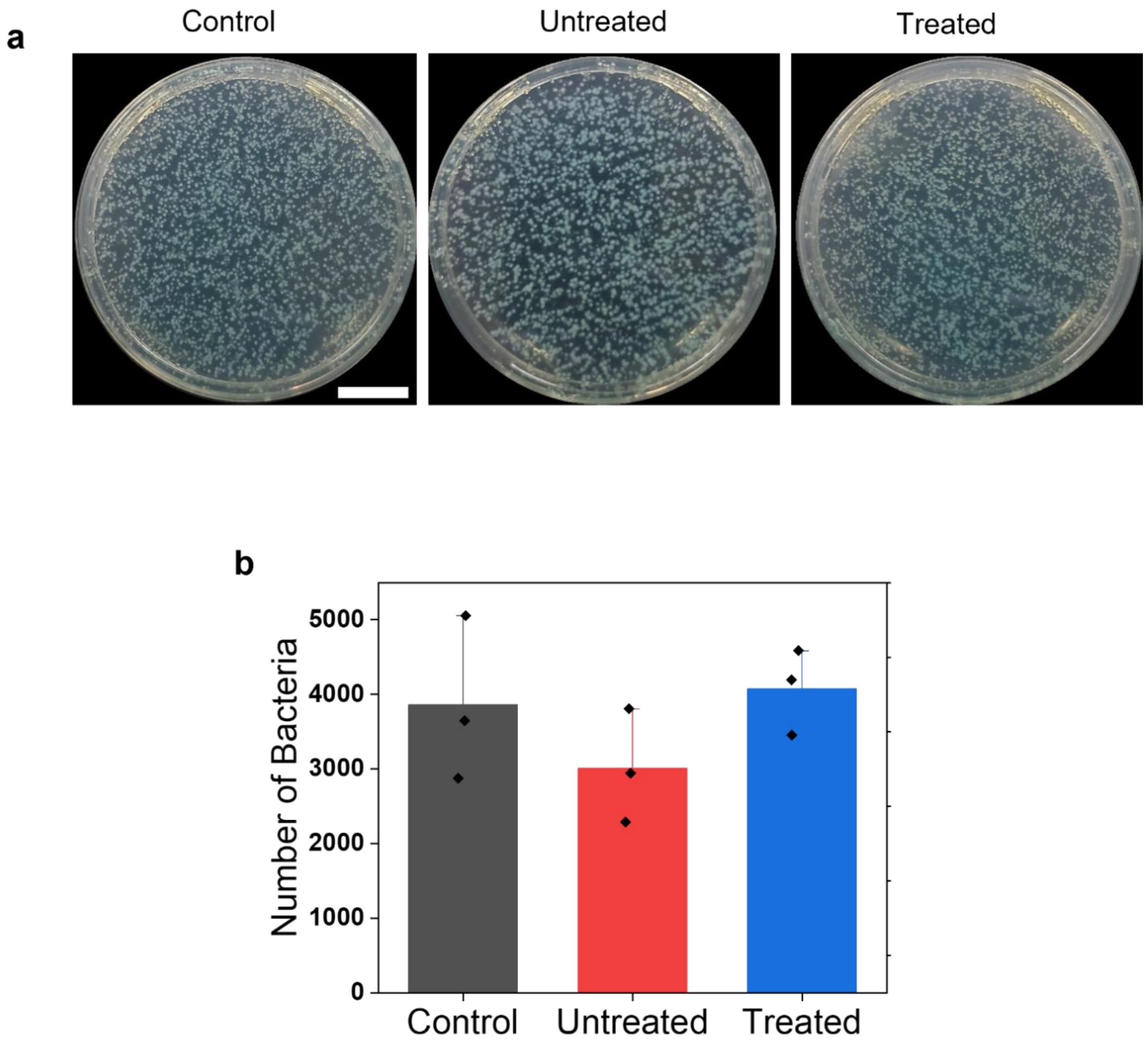

Fig. S28. (a) MRSA was cultured in control, untreated, and treated environments for 24 hours in an agar plate. (b) There was no statistical significance in colony formation. All data are represented as mean ± SD.
